# Supplementary material for: Optical manipulation of sphingolipid biosynthesis using photoswitchable ceramides
Source: eLife. 2019 Feb 5;8:e43230. doi: 10.7554/eLife.43230 (PMC6386522; doi:10.7554/eLife.43230)

Optical manipulation of sphingolipid biosynthesis using photoswitchable ceramides

Matthijs Kol[$1], Ben Williams[$2], Henry Toombs-Ruane[2], Henri G. Franquelim[3], Sergei Korneev[1], Christian Schroeer[1], Petra Schwille[3], Dirk Trauner*[2,4], Joost C.M. Holthuis*[1,5], James Allen Frank*[2,6]

[1]Molecular Cell Biology Division, Department of Biology/Chemistry, University of Osnabrück, 49076 Osnabrück, Germany. [2]Department of Chemistry and Center for Integrated Protein Science, Ludwig Maximilians University Munich, Butenandtstraße 5-13, 81377 Munich, Germany. [3]Department of Cellular and Molecular Biophysics, Max Planck Institute of Biochemistry, Am Klopferspitz 18, 82152 Martinsried, Germany. [4]Department of Chemistry, New York Univsersity, 100 Washington Square East, New York, NY 10003-6699, USA. [5]Membrane Biochemistry & Biophysics, Bijvoet Center and Institute of Biomembranes, Utrecht University, 3584 CH Utrecht, the Netherlands. [6]*present address:* The Vollum Institute, Oregon Health & Science University, 3181 S.W. Sam Jackson Park Rd., Portland, Oregon 97239, USA

[$]these authors contributed equally to this work
*co-corresponding authors

James Allen Frank – frankja@ohsu.edu
Joost C.M. Holthuis - joost.holthuis@biologie.uni-osnabrueck.de
Dirk Trauner – dirktrauner@nyu.edu

Supplementary File 1 - NMR Spectra

^{1}H NMR of **1-(but-3-yn-1-yl)-4-nitrobenzene (2)**

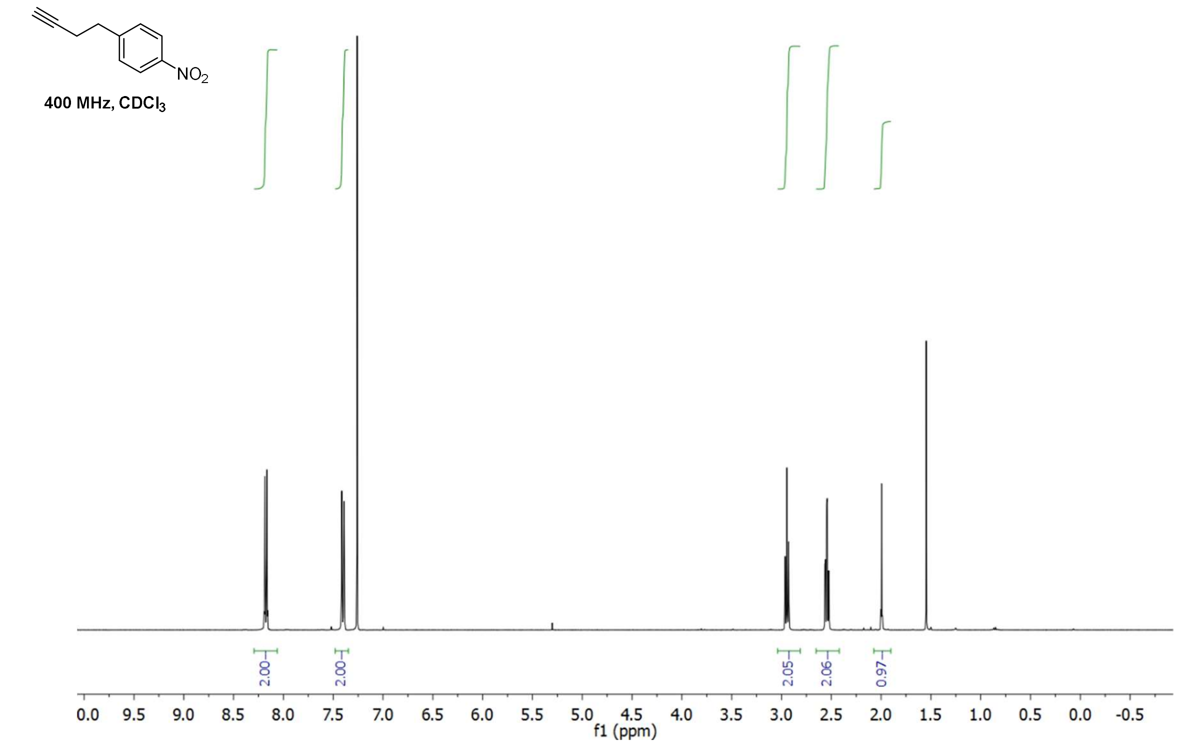

^{13}C NMR of **1-(but-3-yn-1-yl)-4-nitrobenzene (2)**

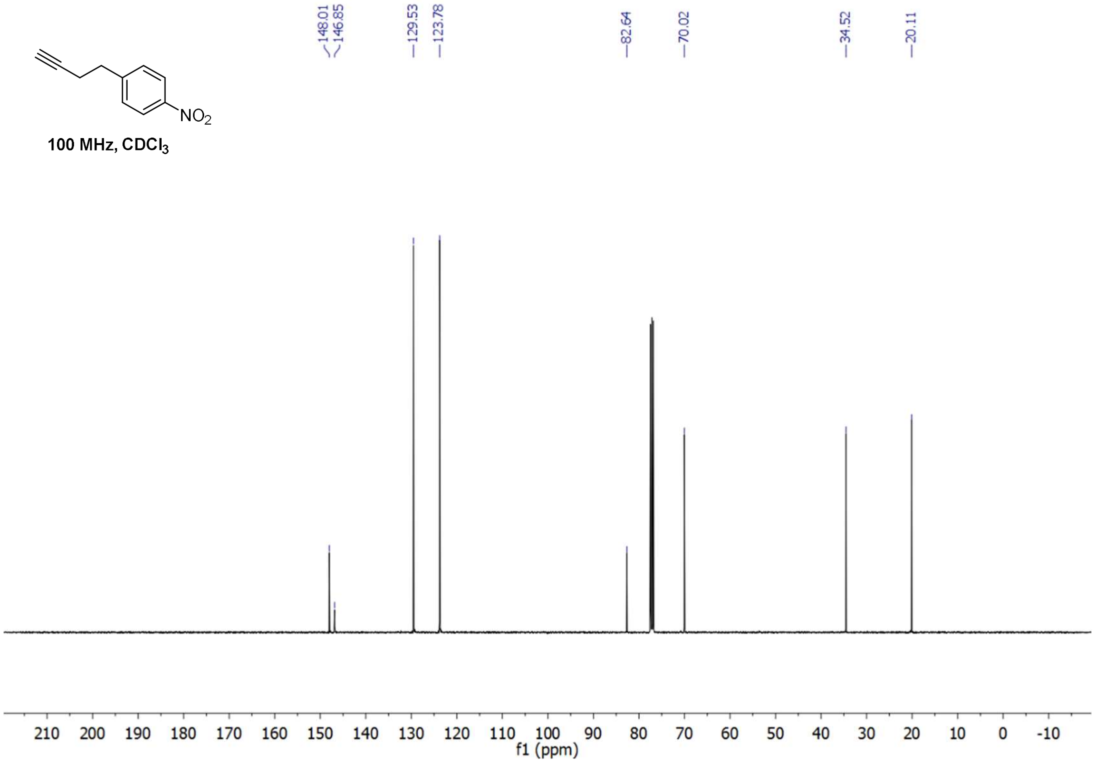

^{1}H NMR of **4-(but-3-yn-1-yl)aniline (3)**

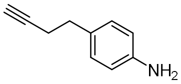

400 MHz, $CDCl_3$

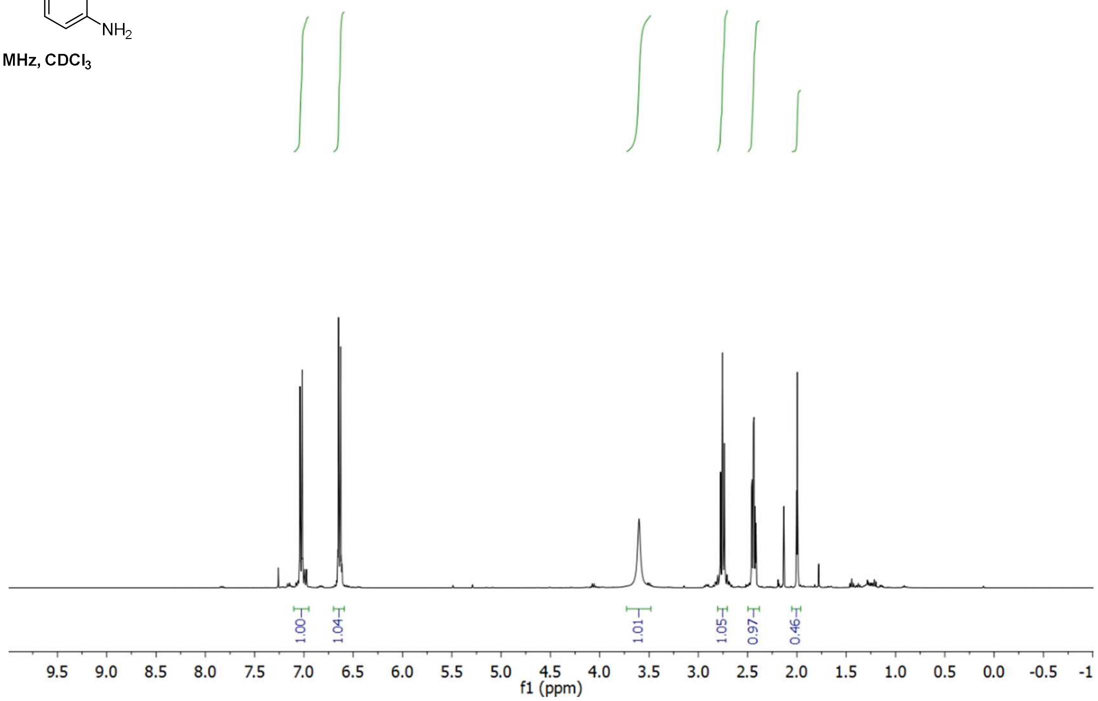

^{13}C NMR of **4-(but-3-yn-1-yl)aniline (3)**

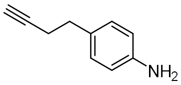

100 MHz, $CDCl_3$

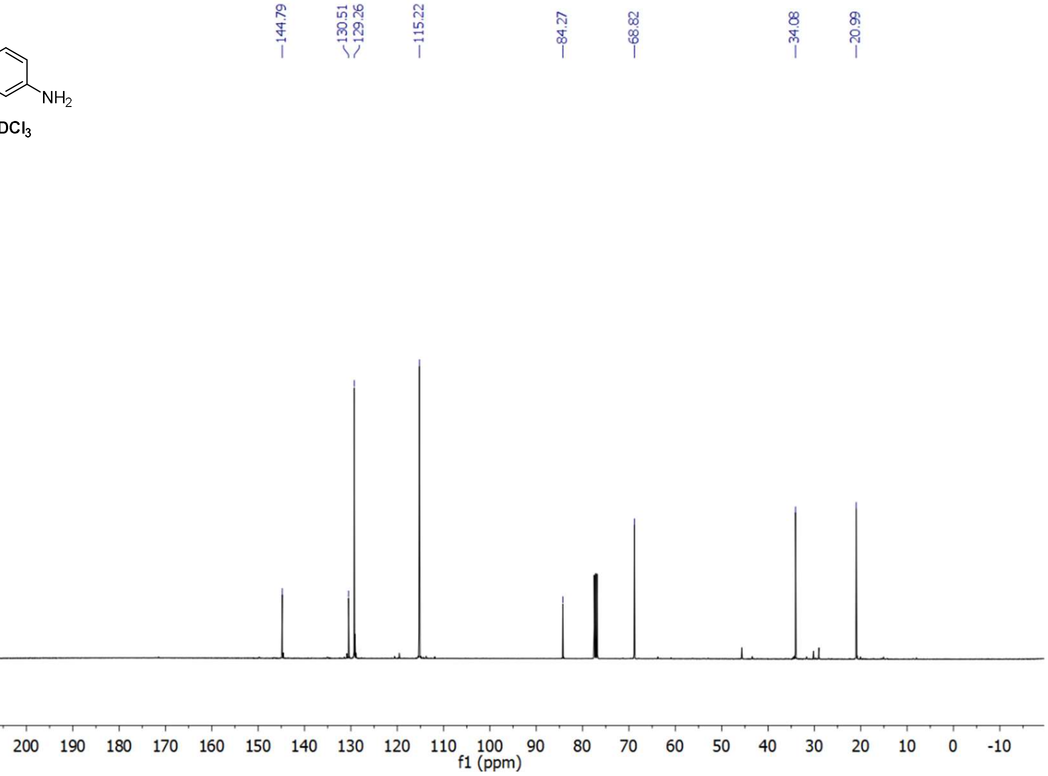

^{1}H NMR of **4-(4-((4-(but-3-yn-1-yl)phenyl)diazenyl)phenyl)butanoic acid (cFAAzo-4)**

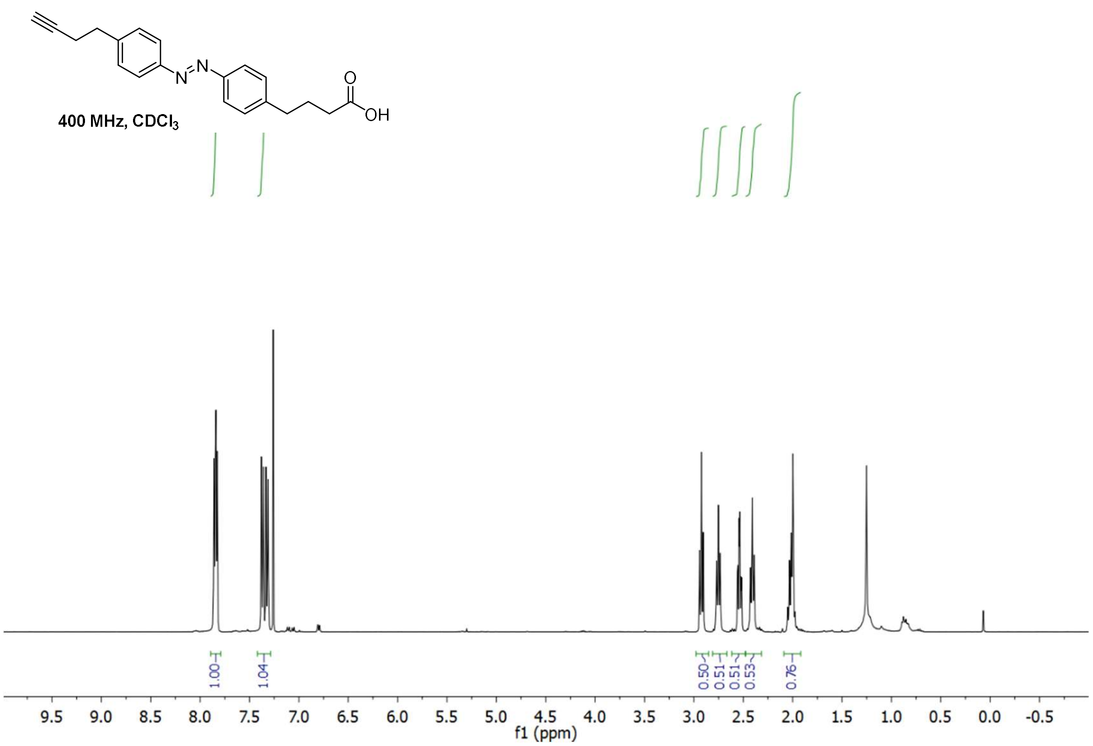

^{13}C NMR of **4-(4-((4-(but-3-yn-1-yl)phenyl)diazenyl)phenyl)butanoic acid (cFAAzo-4)**

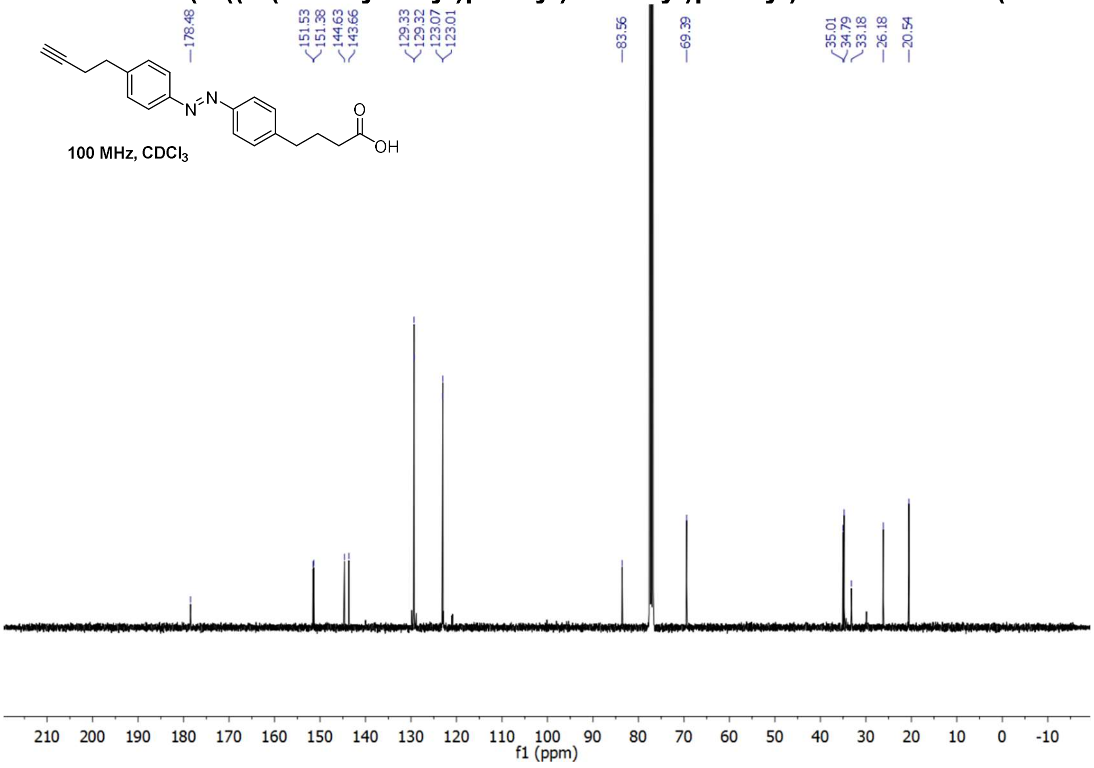

^{1}H NMR of **4-(4-(4-(but-3-yn-1-yl)phenyl)diazenyl)phenyl)-*N*-((2*S*,3*R*,*E*)-1,3-dihydroxyoctadec-4-en-2-yl)butanamide (caCer-1)**

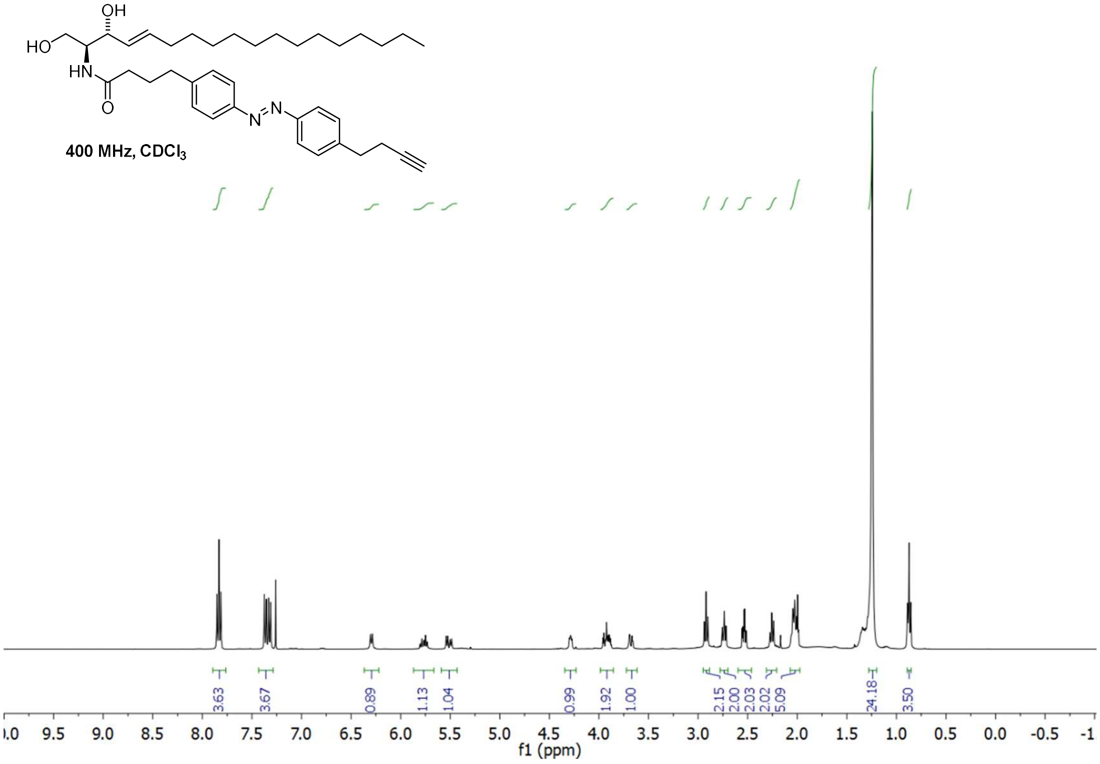

^{13}C NMR of **4-(4-(4-(but-3-yn-1-yl)phenyl)diazenyl)phenyl)-*N*-((2*S*,3*R*,*E*)-1,3-dihydroxyoctadec-4-en-2-yl)butanamide (caCer-1)**

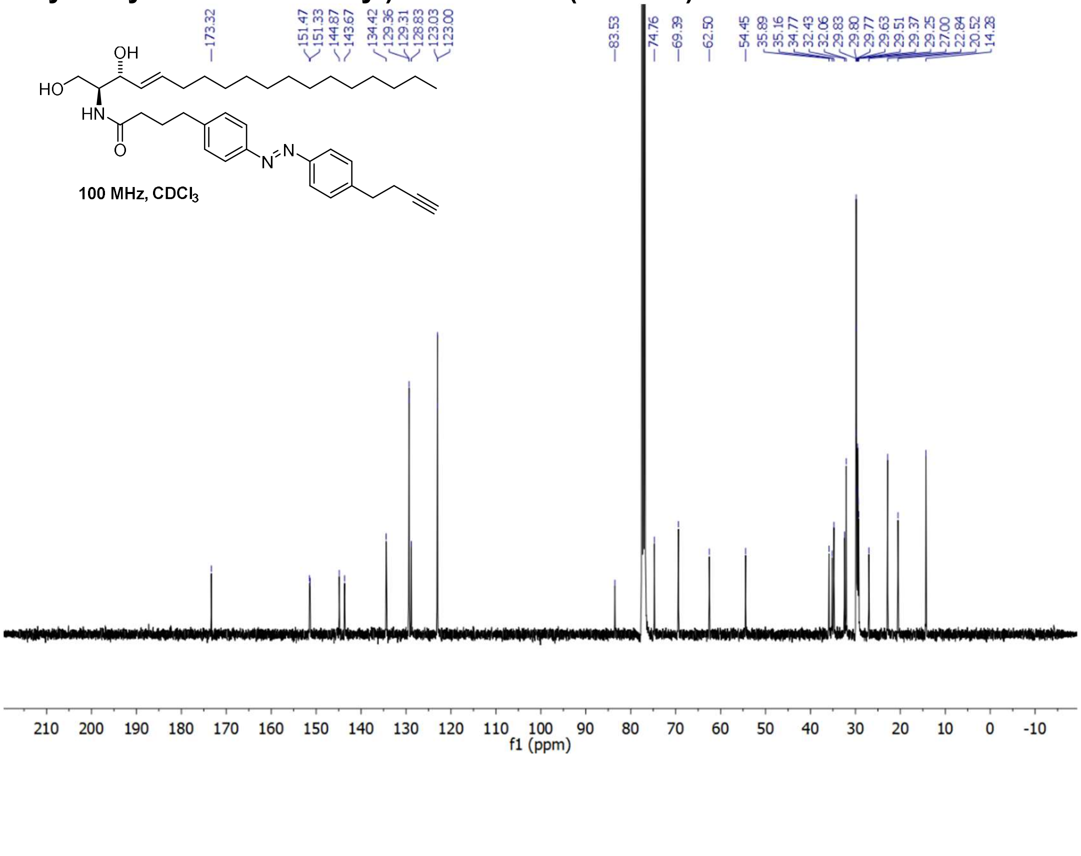

^{1}H NMR of **4-((4-(but-3-yn-1-yl)phenyl)diazenyl)benzoic acid (cFAAzo-1)**

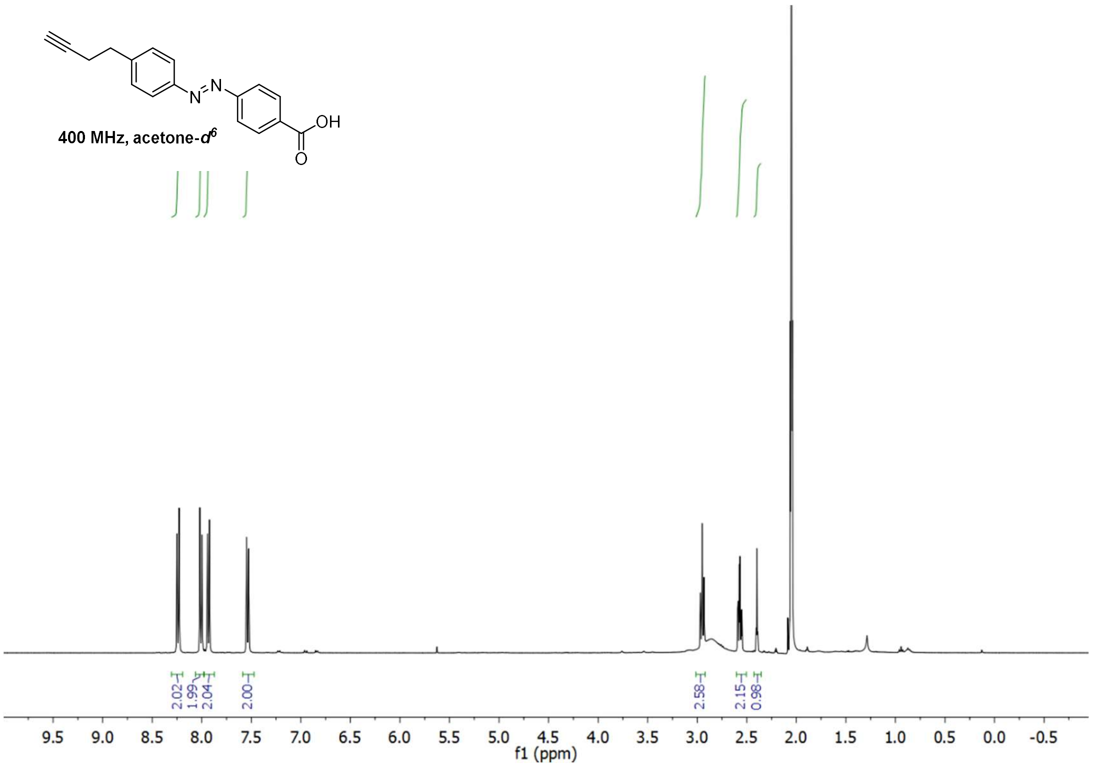

^{13}C NMR of **4-((4-(but-3-yn-1-yl)phenyl)diazenyl)benzoic acid (cFAAzo-1)**

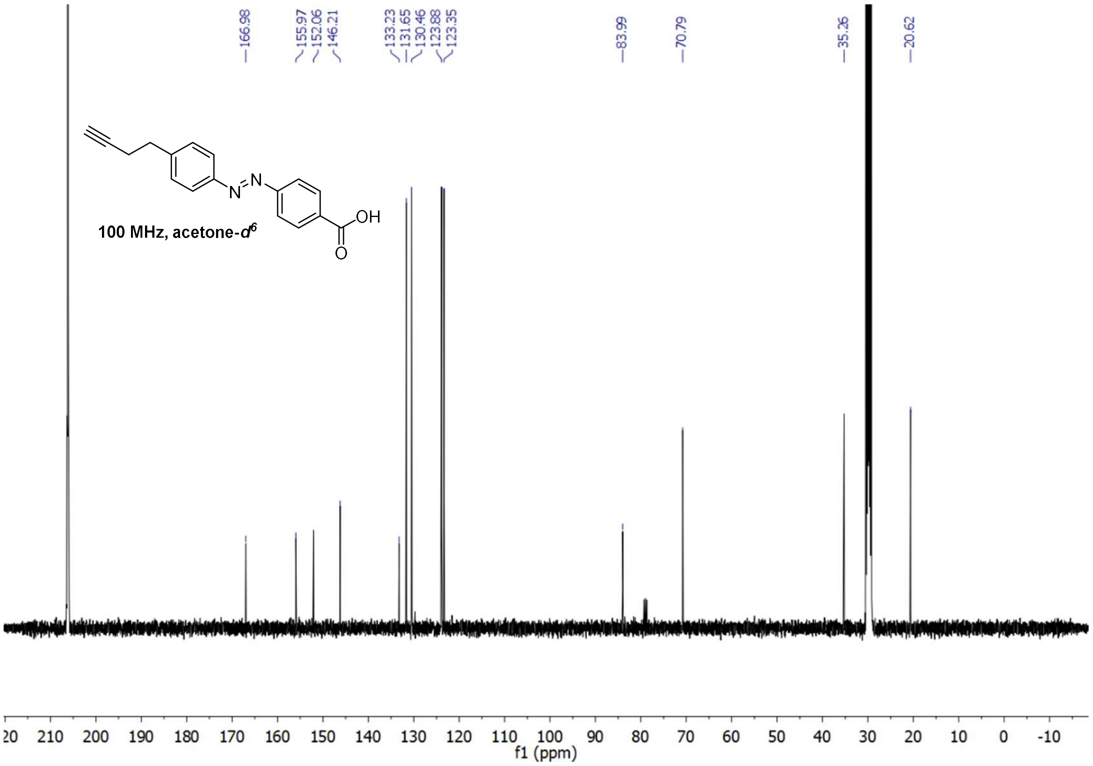

^{1}H NMR of **4-(4-(but-3-yn-1-yl)phenyl)diazenyl-N-((2*S*,3*R*,*E*)-1,3-dihydroxyoctadec-4-en-2-yl)benzamide (caCer-2)**

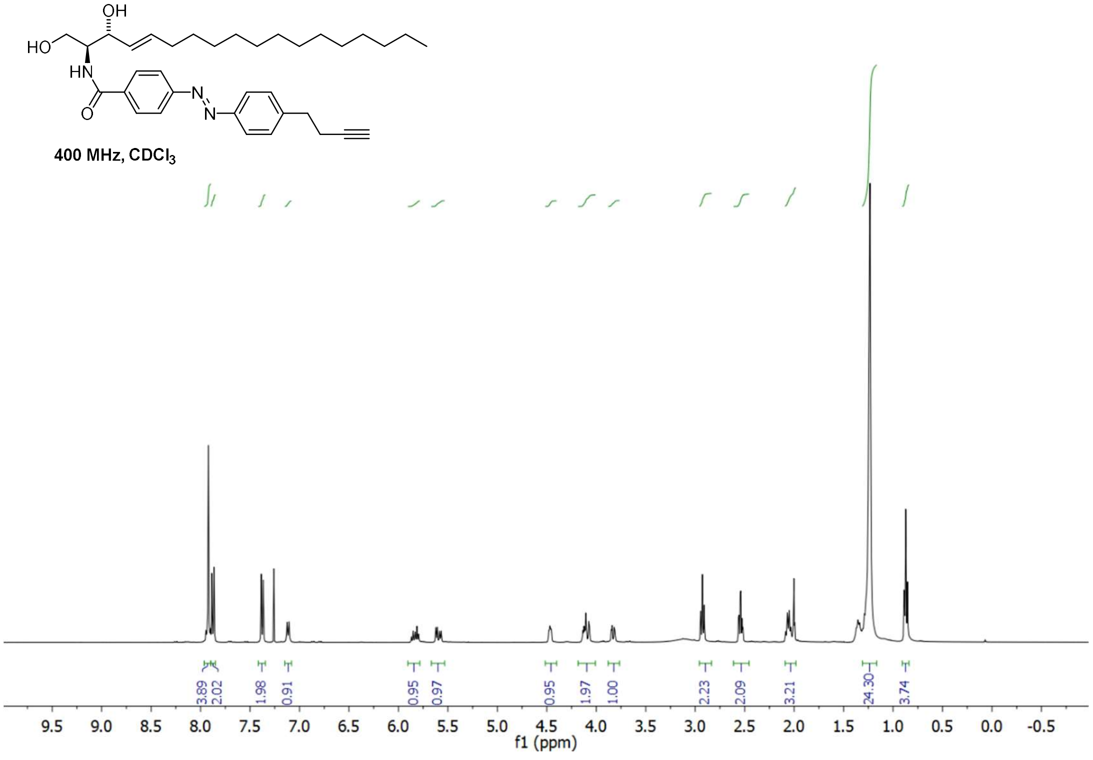

^{13}C NMR of **4-(4-(but-3-yn-1-yl)phenyl)diazenyl-N-((2*S*,3*R*,*E*)-1,3-dihydroxyoctadec-4-en-2-yl)benzamide (caCer-2)**

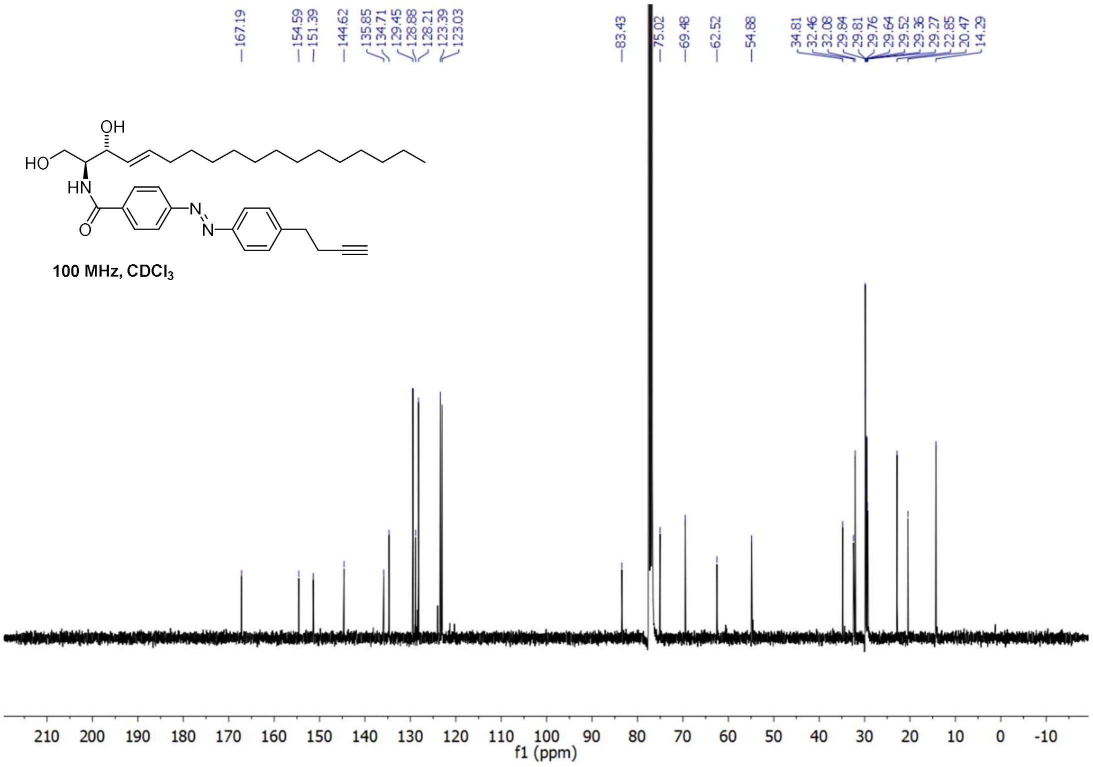

^{1}H NMR of **pentadec-7-yn-1-ol (S1)**

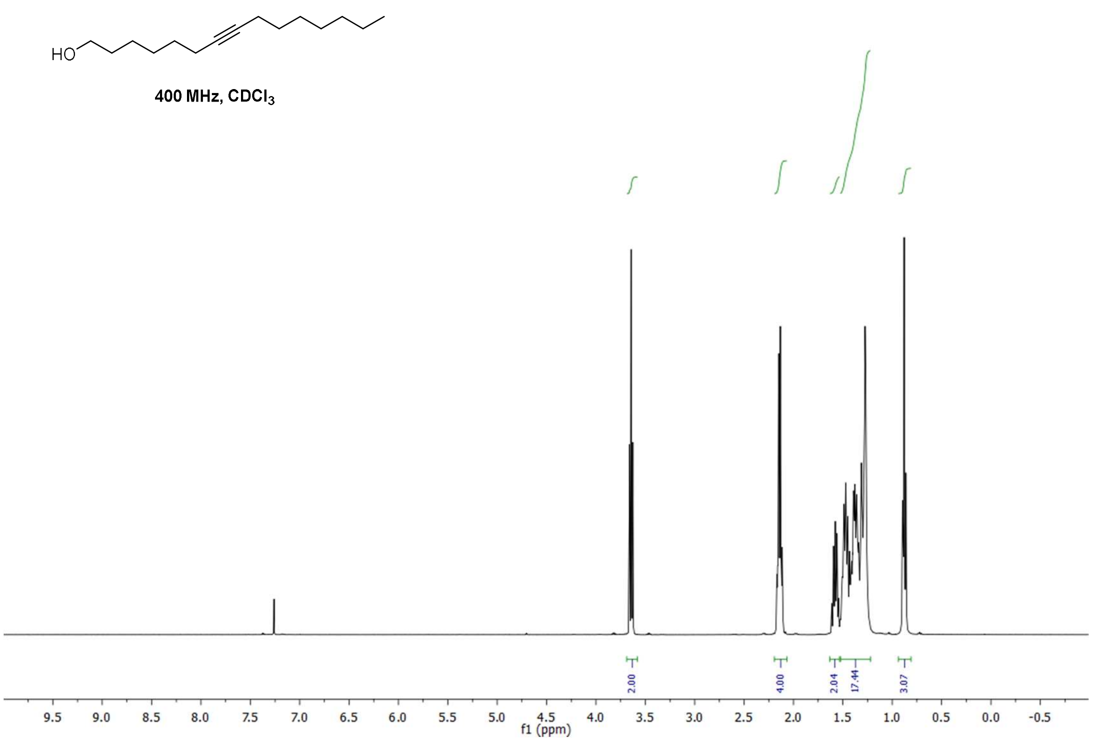

^{13}C NMR of **pentadec-7-yn-1-ol (S1)**

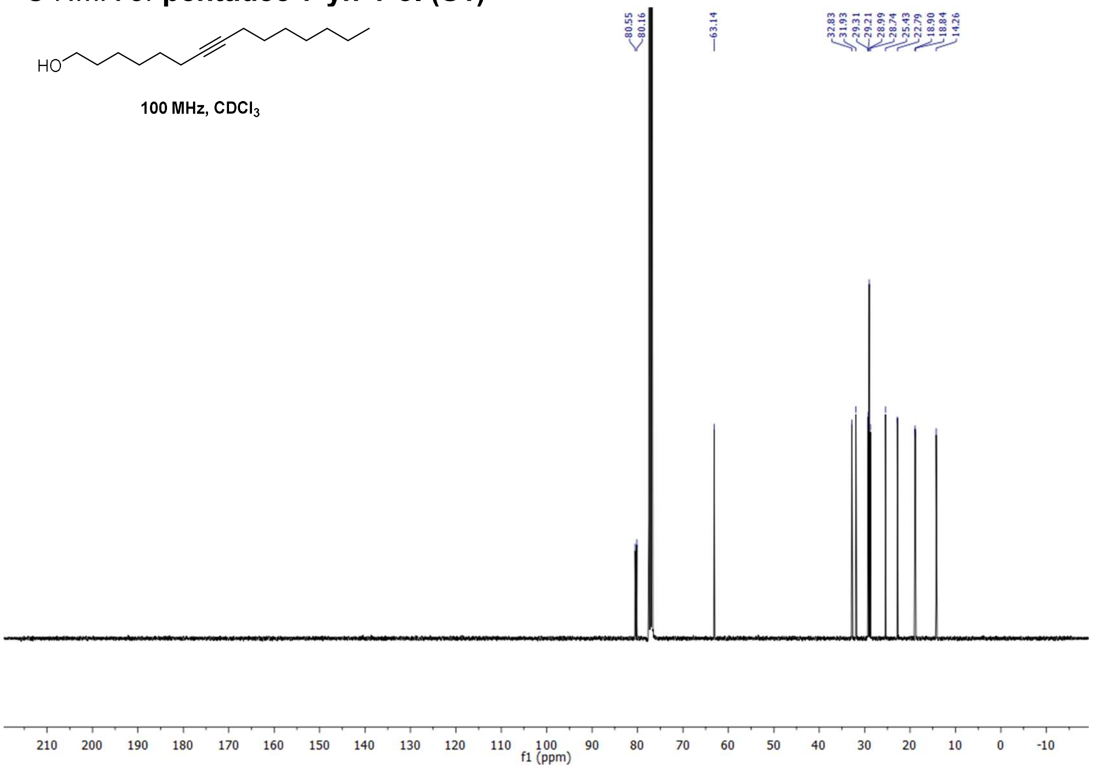

^{1}H NMR of **pentadec-14-yn-1-ol (S2)**

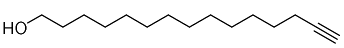

400 MHz, $CDCl_3$

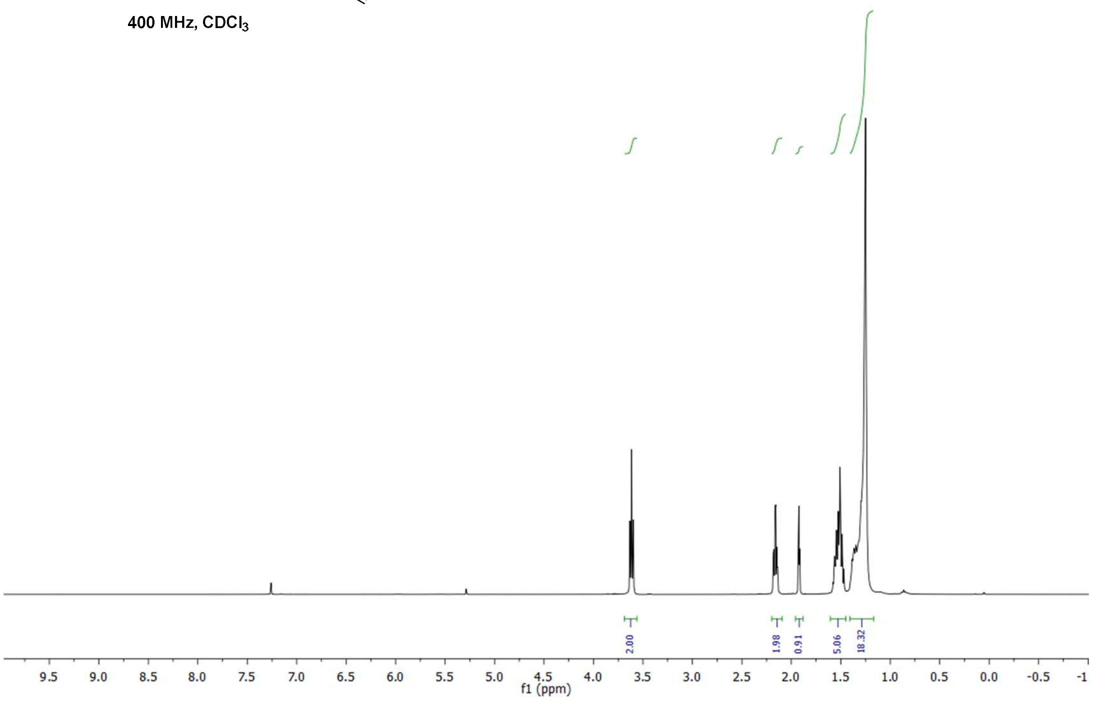

^{1}H NMR of **pentadec-14-ynoic acid (4)**

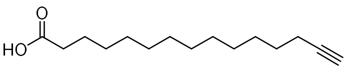

400 MHz, $CDCl_3$

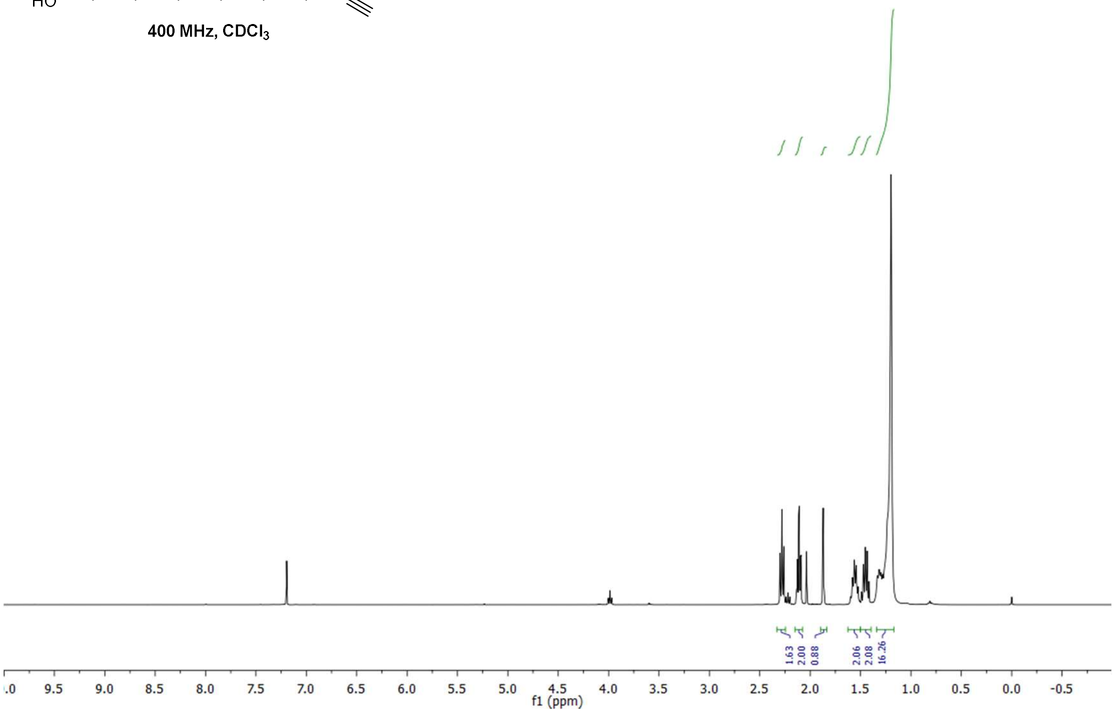

^{1}H NMR of **4-((4-propylphenyl)diazenyl)benzyl alcohol (5)**

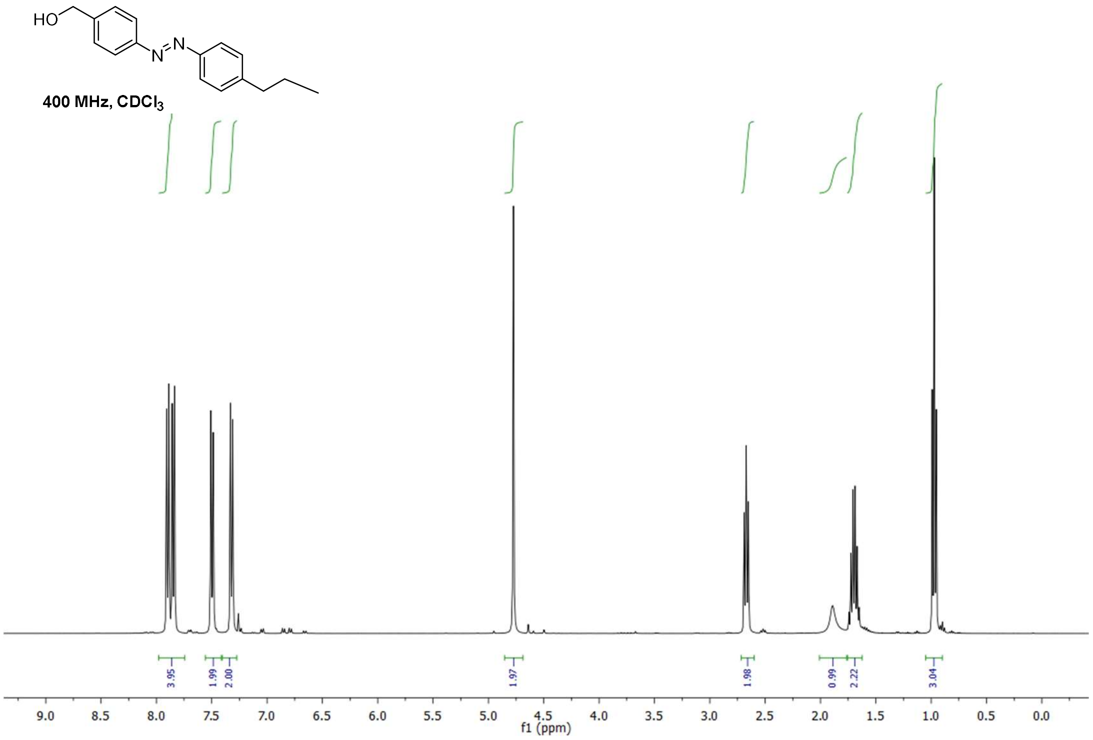

^{13}C NMR of **4-((4-propylphenyl)diazenyl)benzyl alcohol (5)**

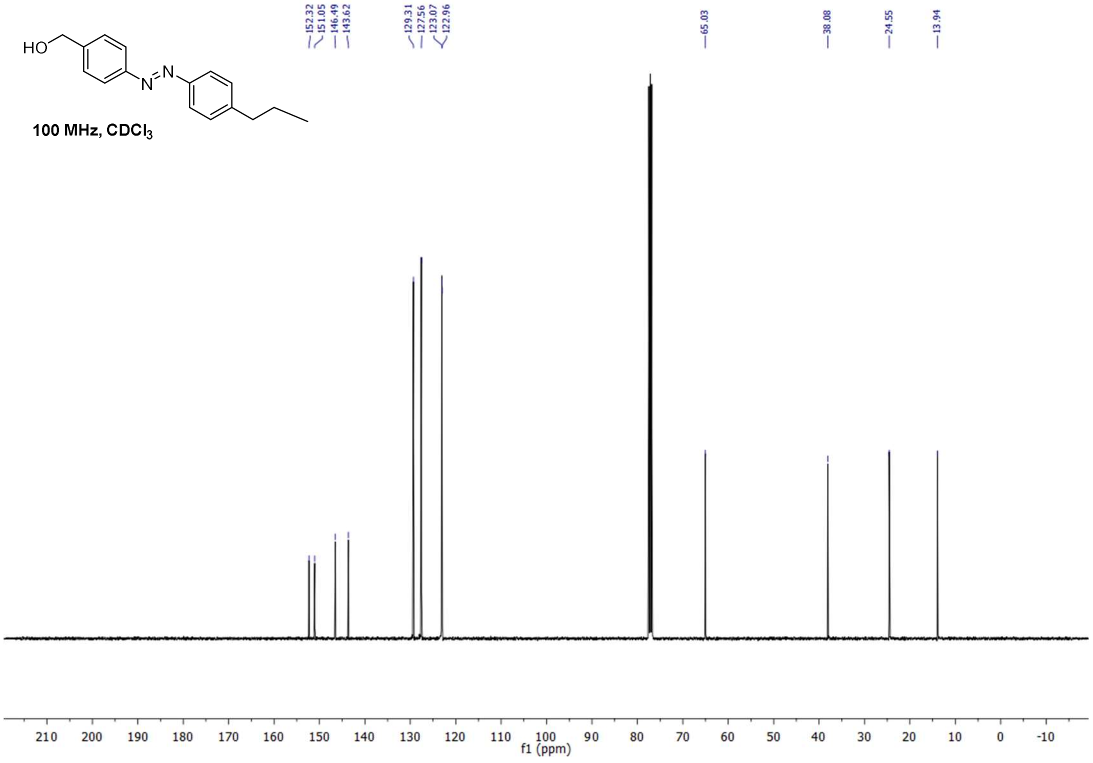

^{1}H NMR of **4-((4-propylphenyl)diazenyl)benzaldehyde (6)**

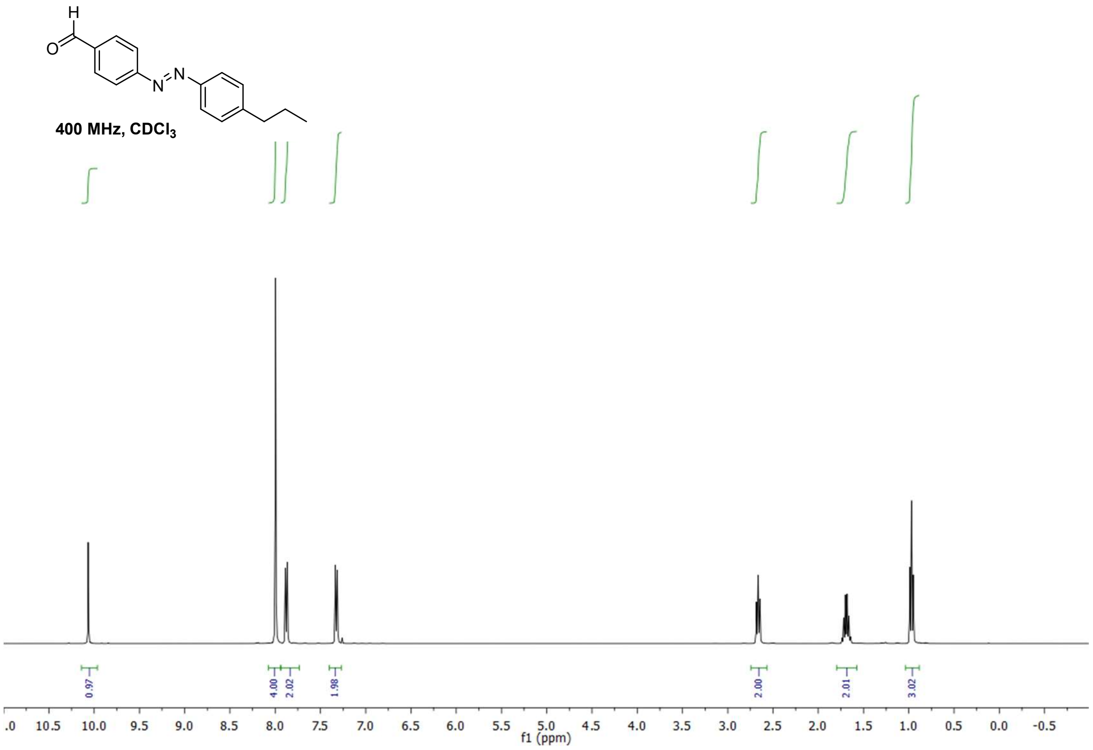

^{13}C NMR of **4-((4-propylphenyl)diazenyl)benzaldehyde (6)**

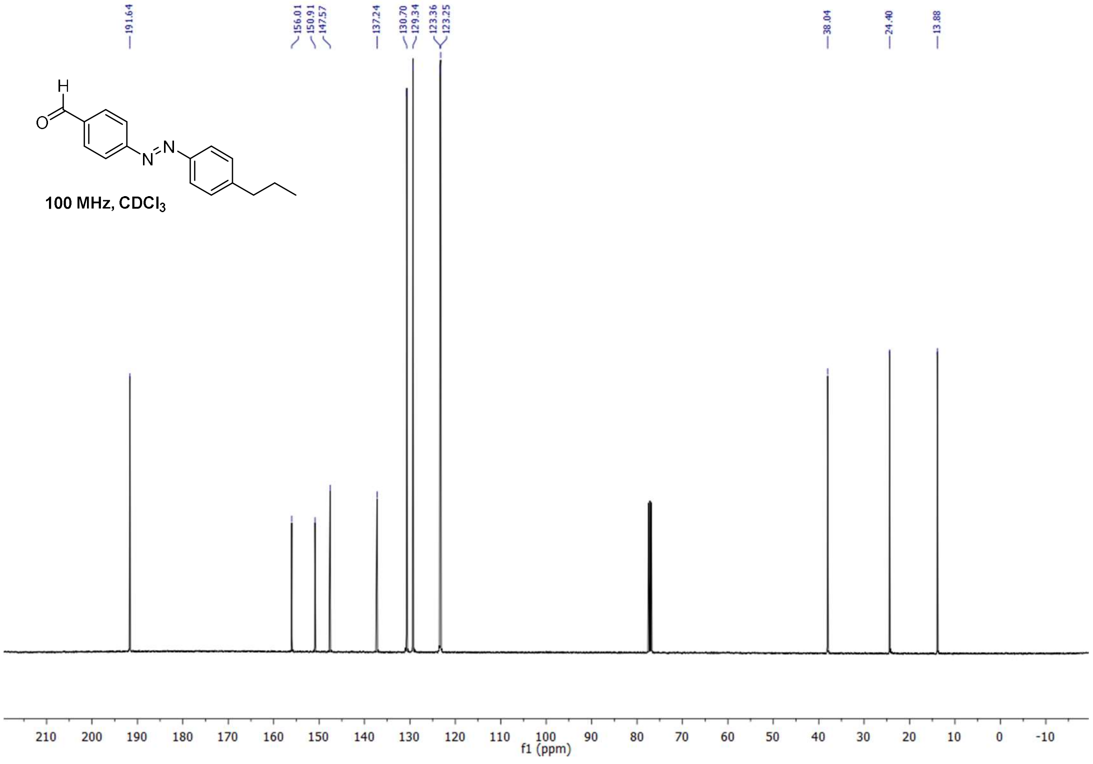

^{1}H NMR of **1-(4-propylphenyl)-2-(4-vinylphenyl)diazene (7)**

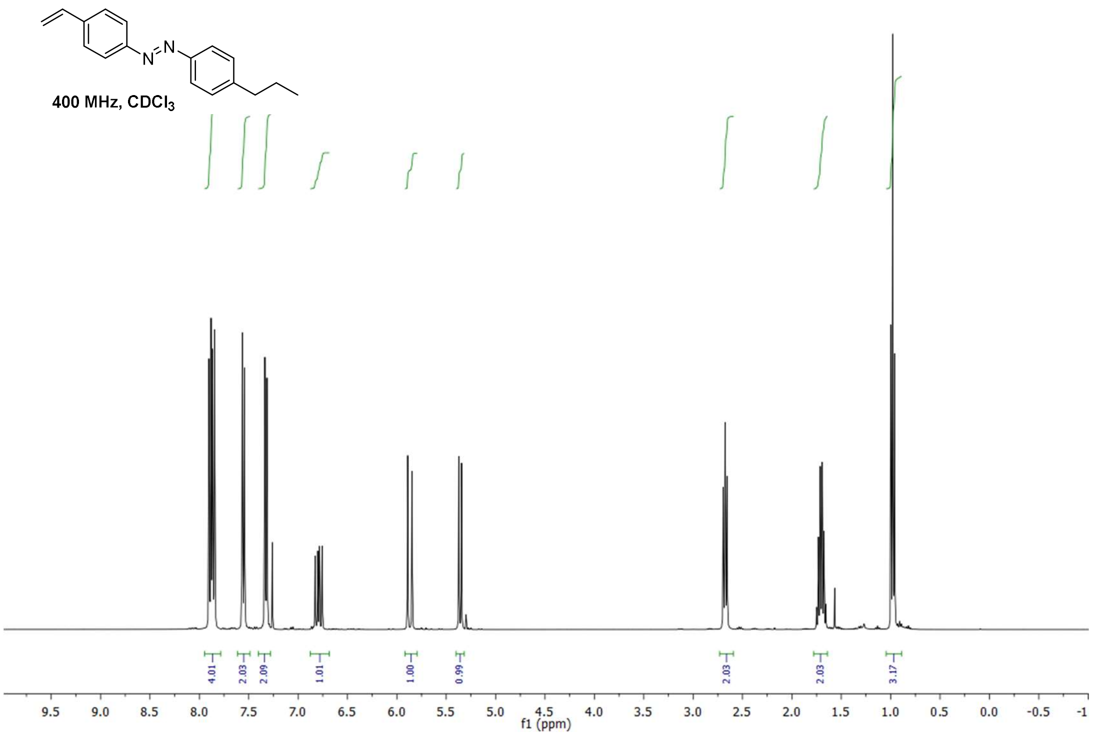

^{13}C NMR of **1-(4-propylphenyl)-2-(4-vinylphenyl)diazene (7)**

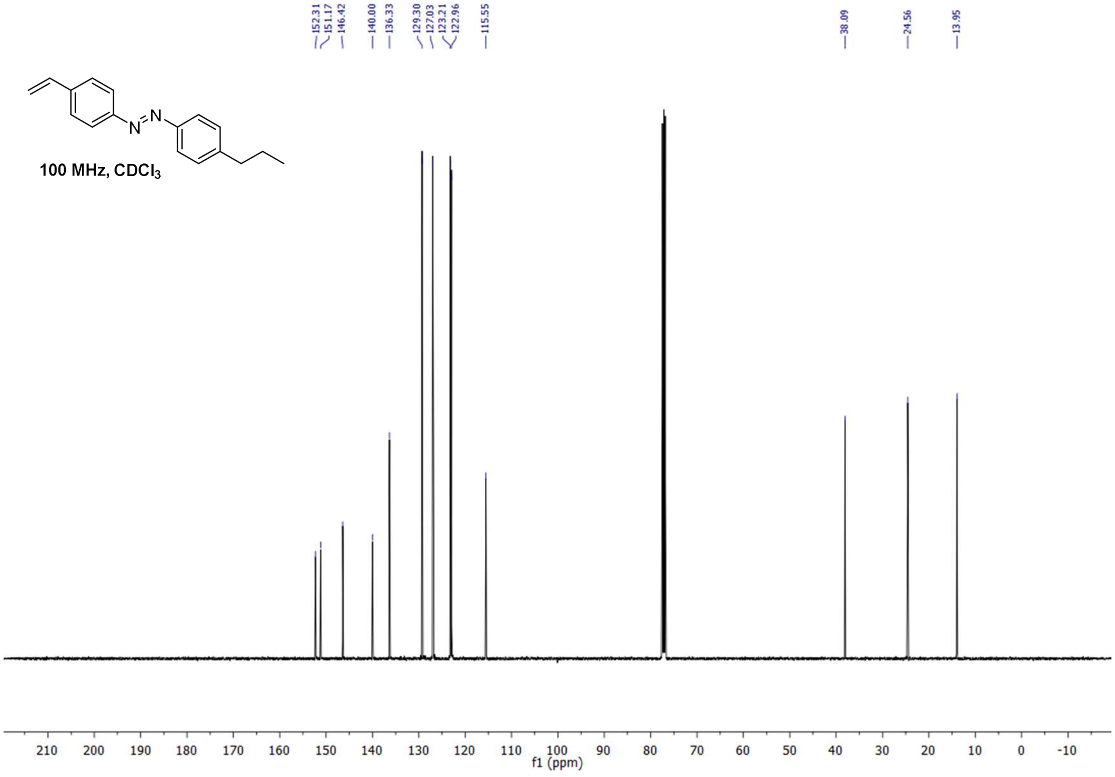

^{1}H NMR of **4-iodo-4’-methylazobenzene (S3)**

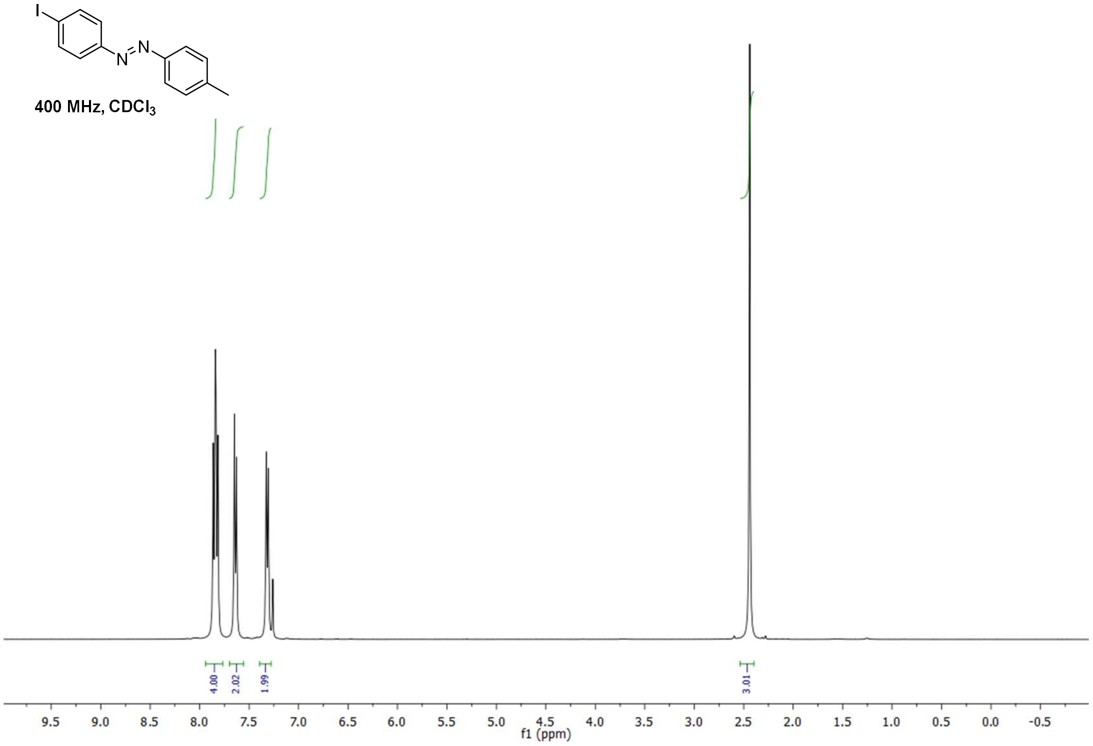

^{13}C NMR of **4-iodo-4’-methylazobenzene (S3)**

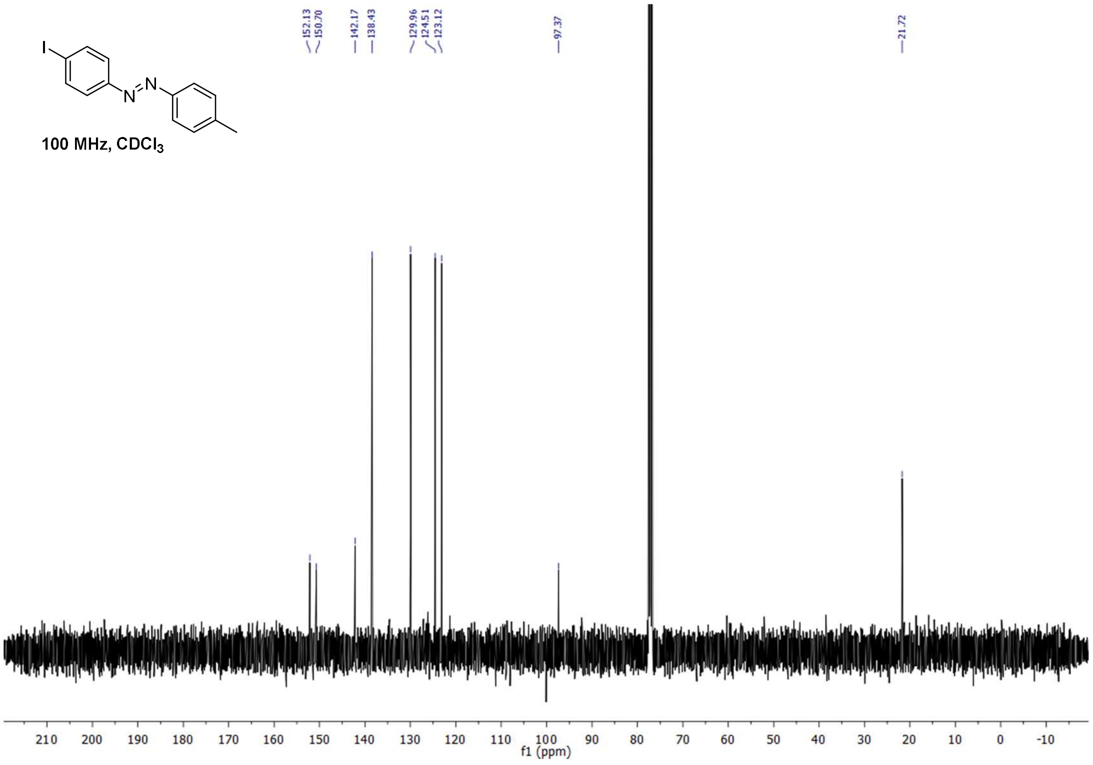

^{1}H NMR of **3-(4-(*p*-tolyldiazenyl)phenyl)propanal (S4)**

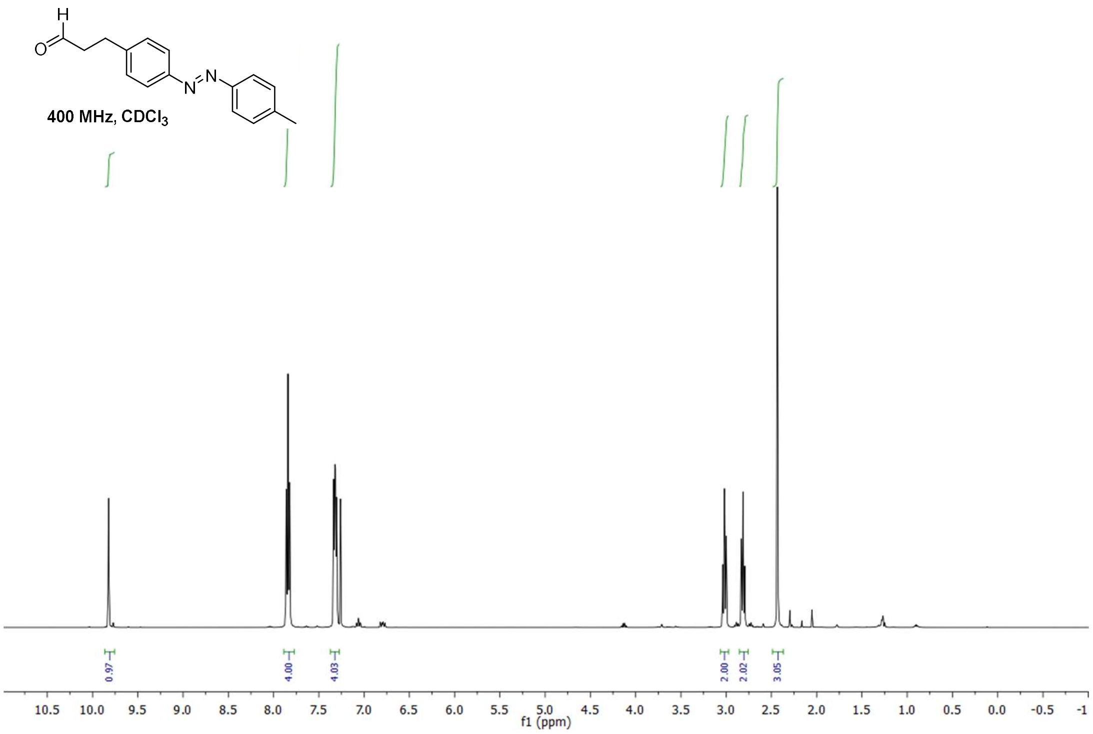

^{13}C NMR of **3-(4-(*p*-tolyldiazenyl)phenyl)propanal (S4)**

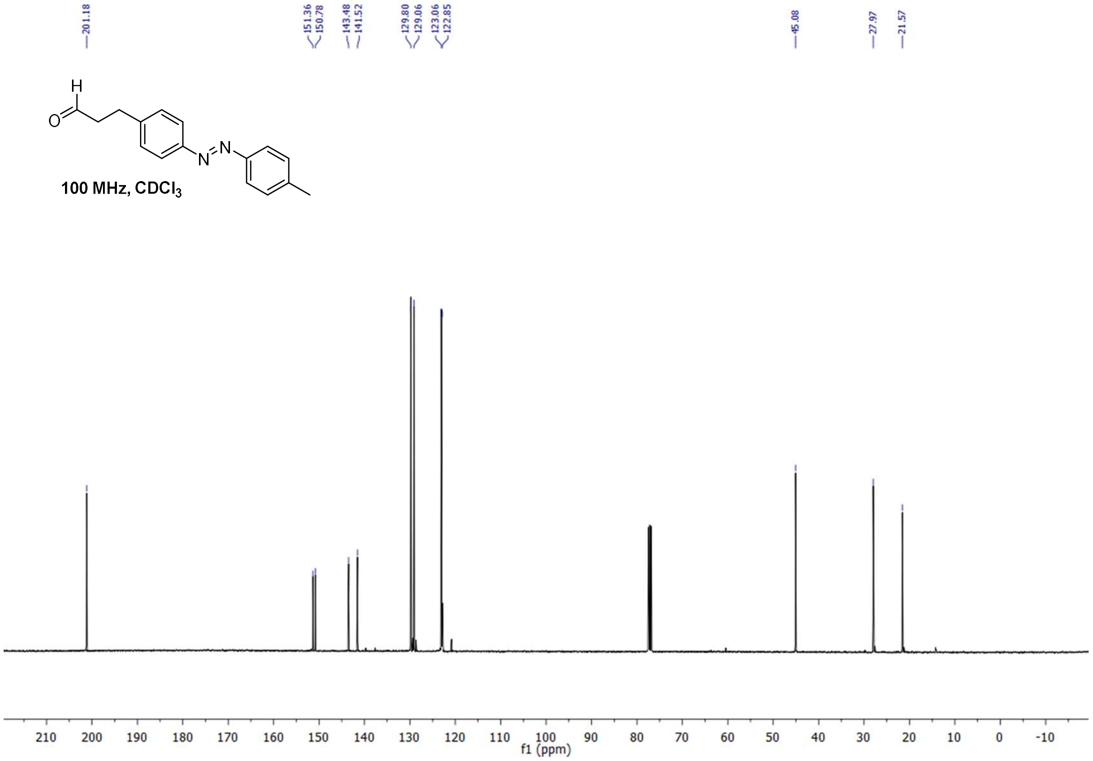

^{1}H NMR of **1-(4-(but-3-en-1-yl)phenyl)-2-(*p*-tolyl)diazene (S5)**

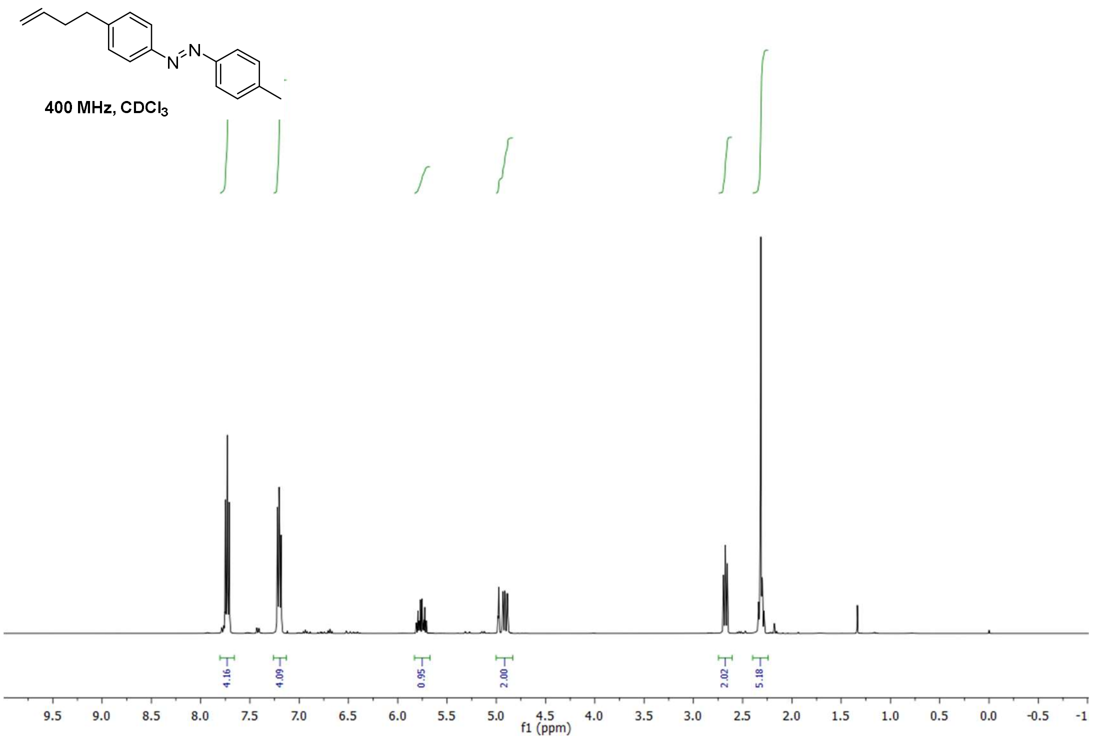

^{13}C NMR of **1-(4-(but-3-en-1-yl)phenyl)-2-(*p*-tolyl)diazene (S5)**

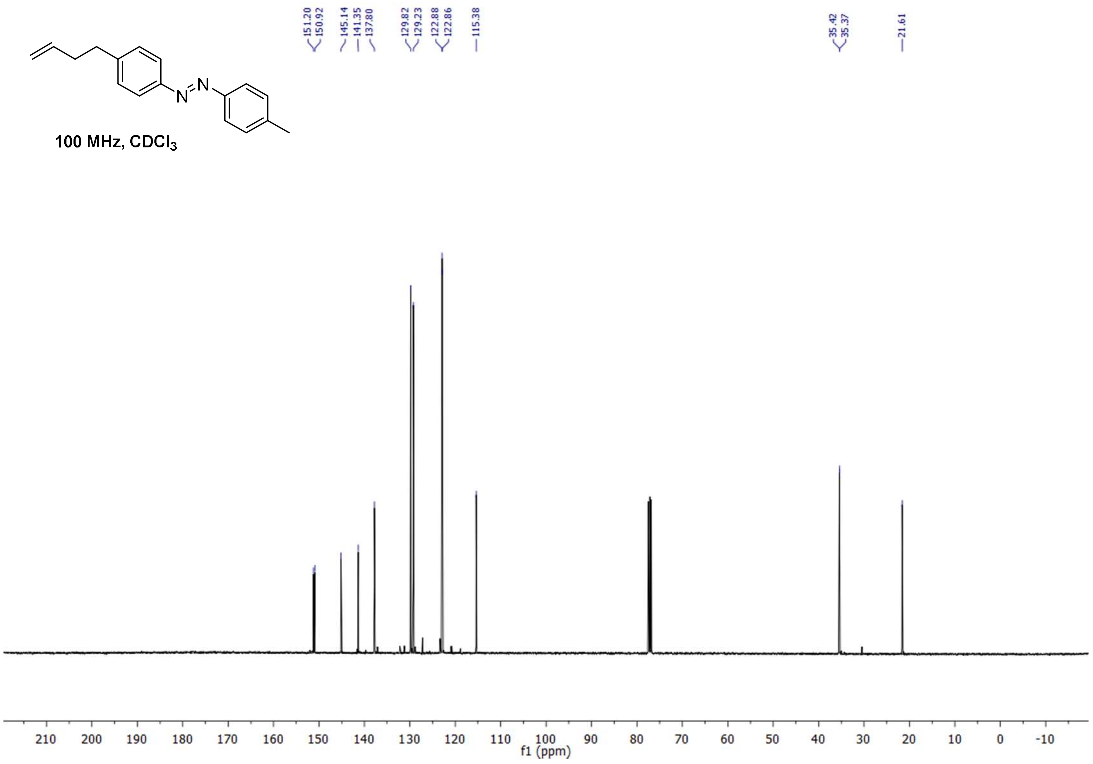

^{1}H NMR of ***N*-Boc-(*R*)-1-((*S*)-2,2-dimethyloxazolidin-4-yl)prop-2-en-1-ol (8)**

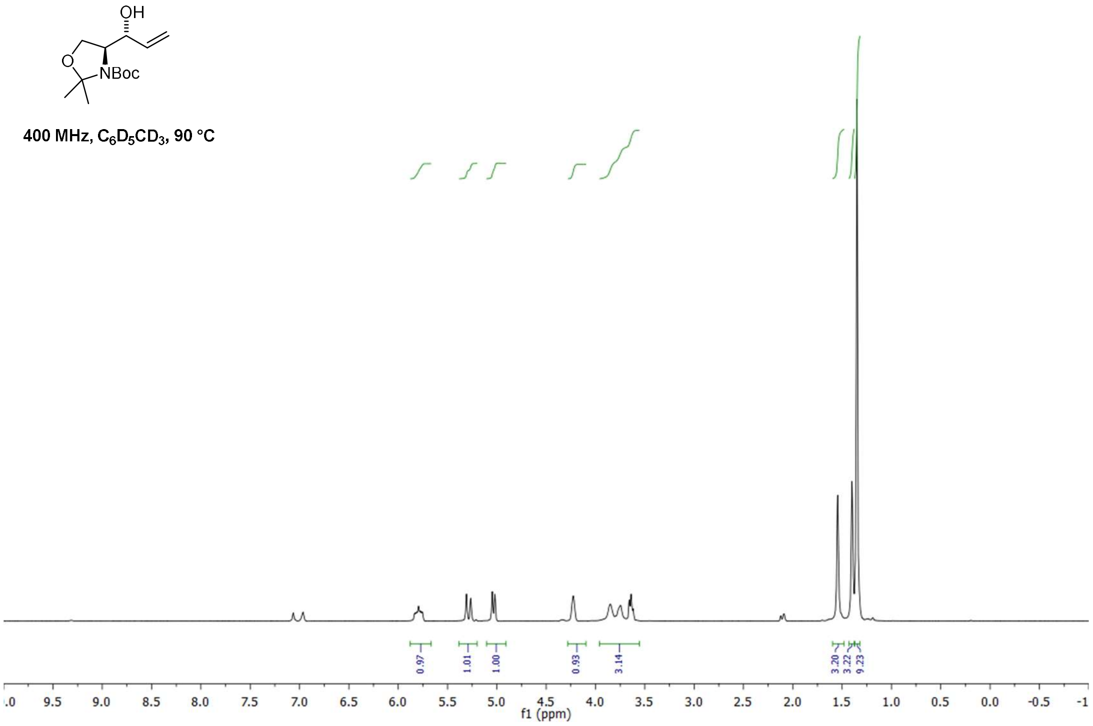

^{13}C NMR of ***N*-Boc-(*R*)-1-((*S*)-2,2-dimethyloxazolidin-4-yl)prop-2-en-1-ol (8)**

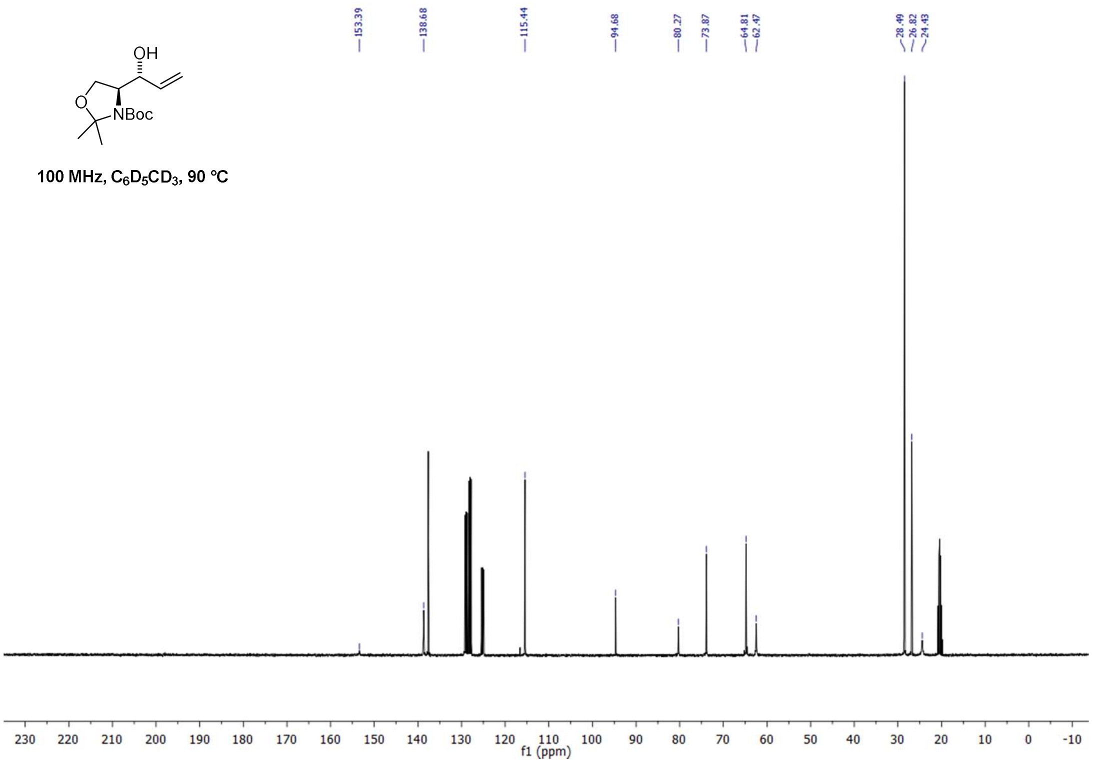

^{1}H NMR of ***N*-Boc-(*R,E*)-1-((*S*)-2,2-dimethyloxazolidin-4-yl)-3-((4-(4-propylphenyl)diazenyl)phenyl)prop-2-en-1-ol (9)**

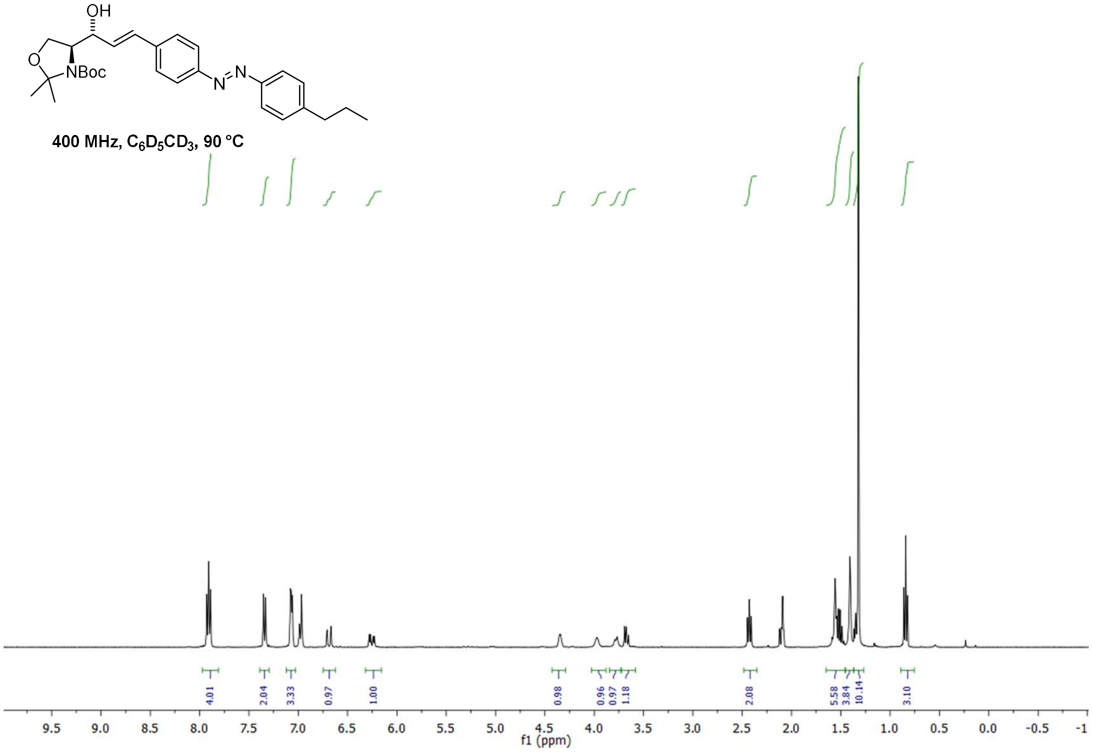

^{13}C NMR of ***N*-Boc-(*R,E*)-1-((*S*)-2,2-dimethyloxazolidin-4-yl)-3-((4-(4-propylphenyl)diazenyl)phenyl)prop-2-en-1-ol (9)**

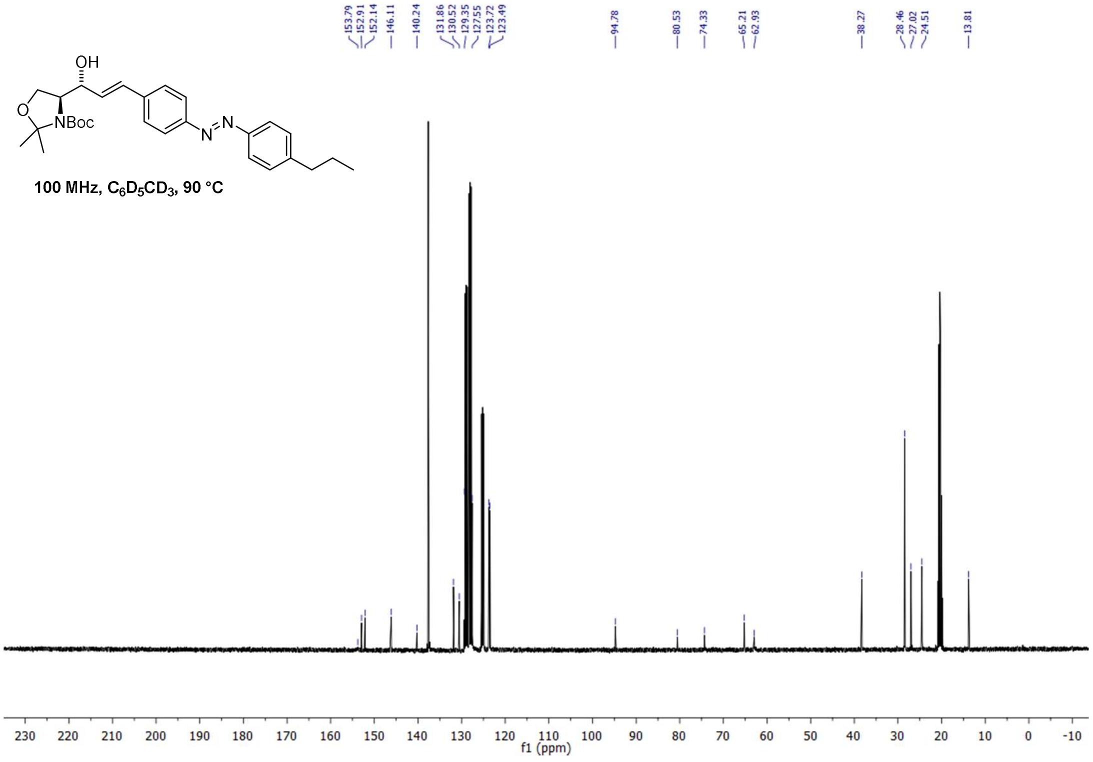

^{1}H NMR of (2*S*,3*R*,*E*)-2-amino-5-(4-((4-propylphenyl)diazenyl)phenyl) pent-4-ene-1,3-diol (aSph-1)

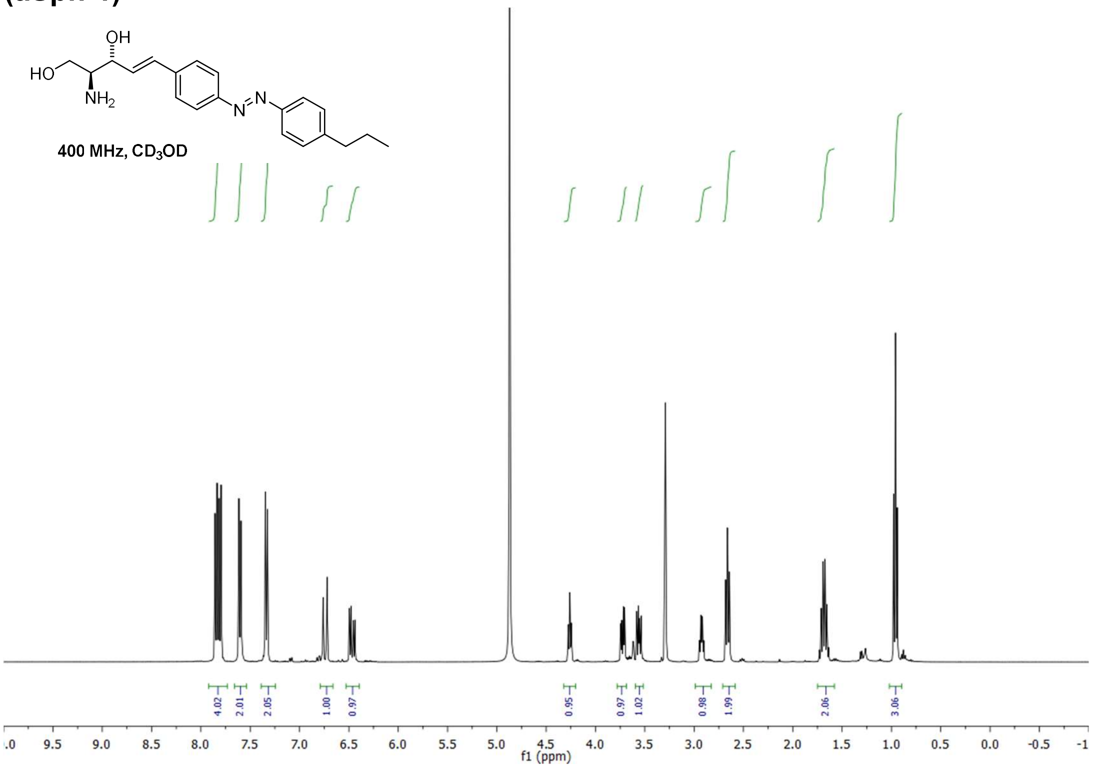

^{13}C NMR of (2*S*,3*R*,*E*)-2-amino-5-(4-((4-propylphenyl)diazenyl)phenyl) pent-4-ene-1,3-diol (aSph-1)

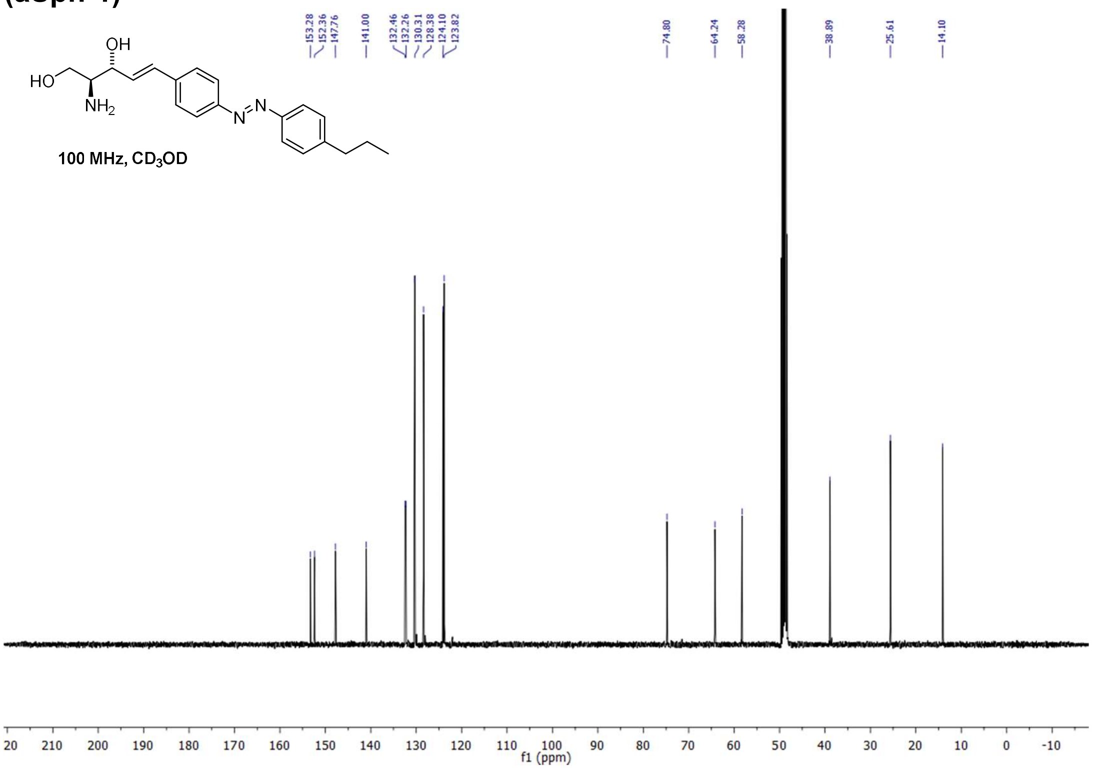

^{1}H NMR of ***N*-((2*S*,3*R*,*E*)-1,3-dihydroxy-5-(4-((4-propylphenyl) diazenyl)phenyl)pent-4-en-2-yl)pentadec-14-ynamide (caCer-3)**

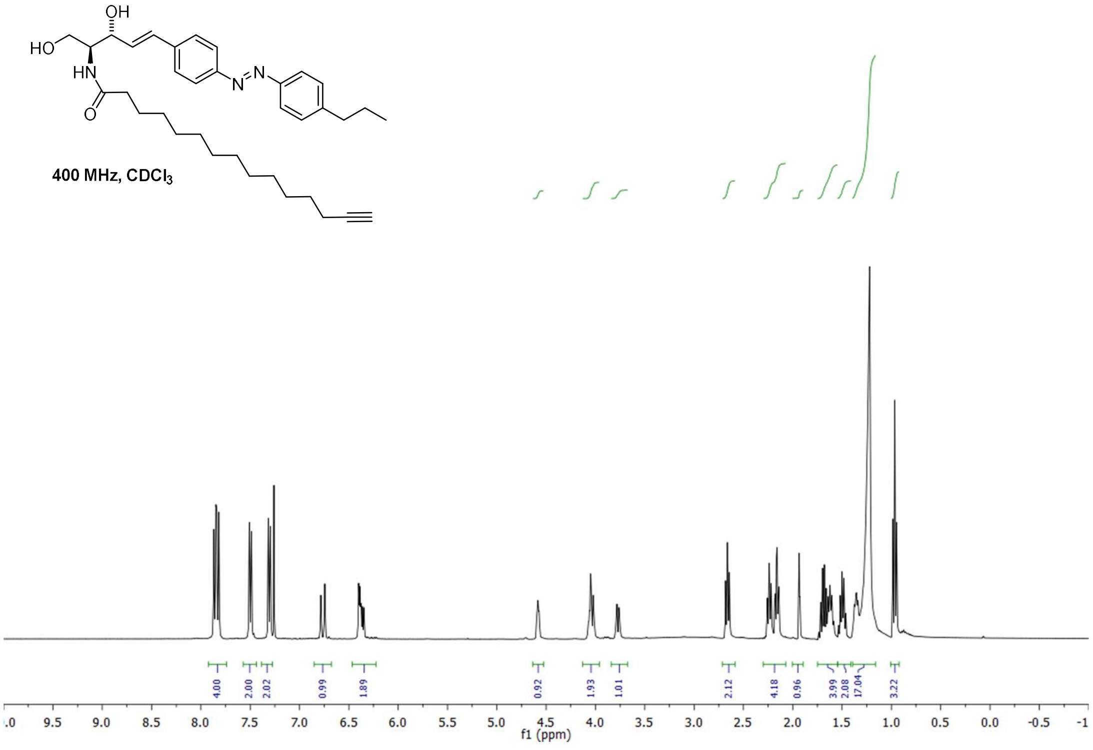

^{13}C NMR of ***N*-((2*S*,3*R*,*E*)-1,3-dihydroxy-5-(4-((4-propylphenyl) diazenyl)phenyl)pent-4-en-2-yl)pentadec-14-ynamide (caCer-3)**

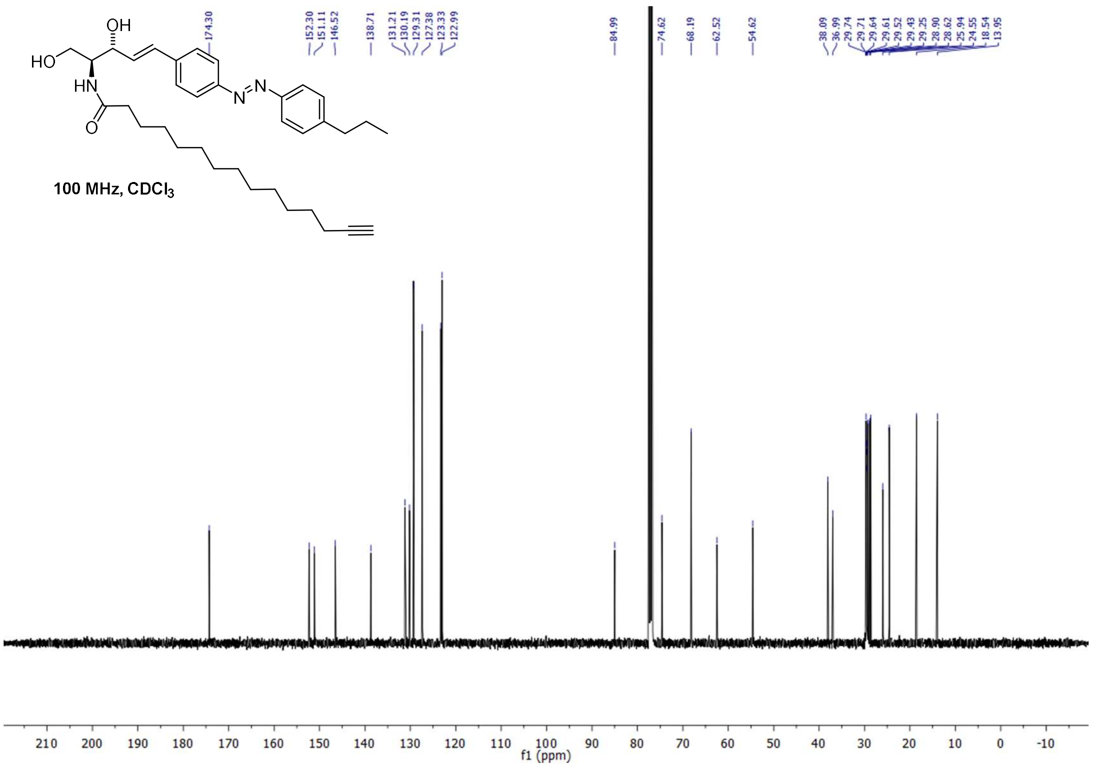

^{1}H NMR of compound ***N*-Boc-(*R,E*)-1-((*S*)-2,2-dimethyloxazolidin-4-yl)-6-(4-(*p*-tolyldiazenyl)phenyl)hex-2-en-1-ol (S6)**

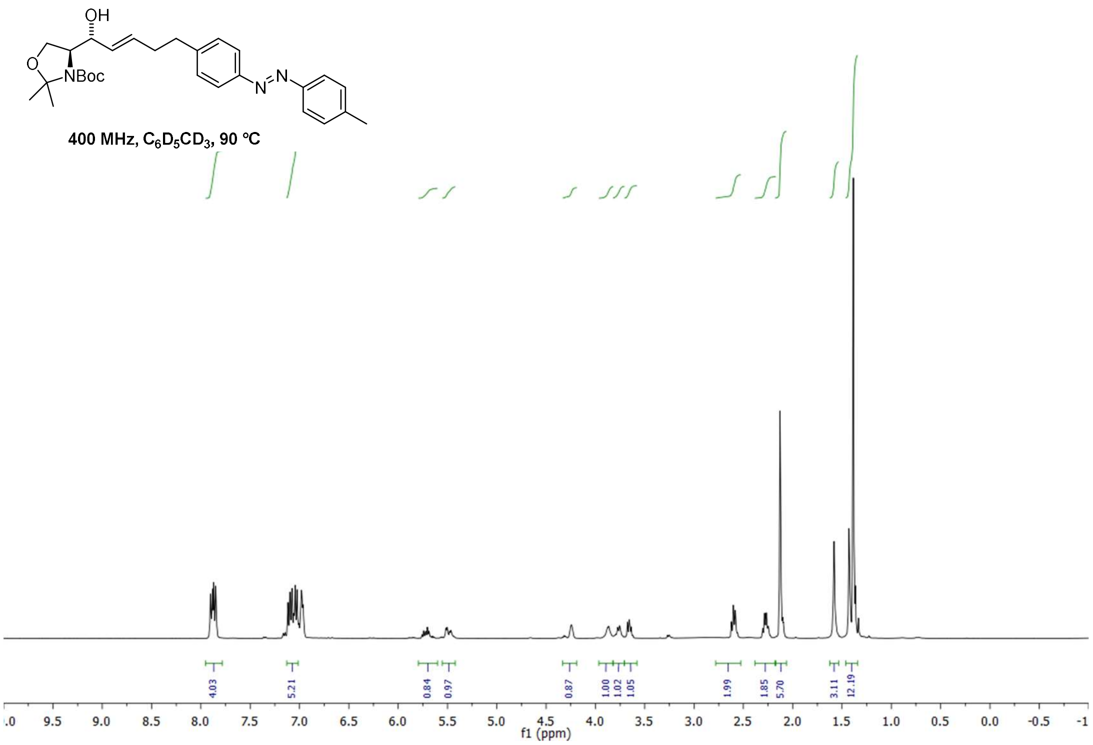

^{13}C NMR of compound ***N*-Boc-(*R,E*)-1-((*S*)-2,2-dimethyloxazolidin-4-yl)-6-(4-(*p*-tolyldiazenyl)phenyl)hex-2-en-1-ol (S6)**

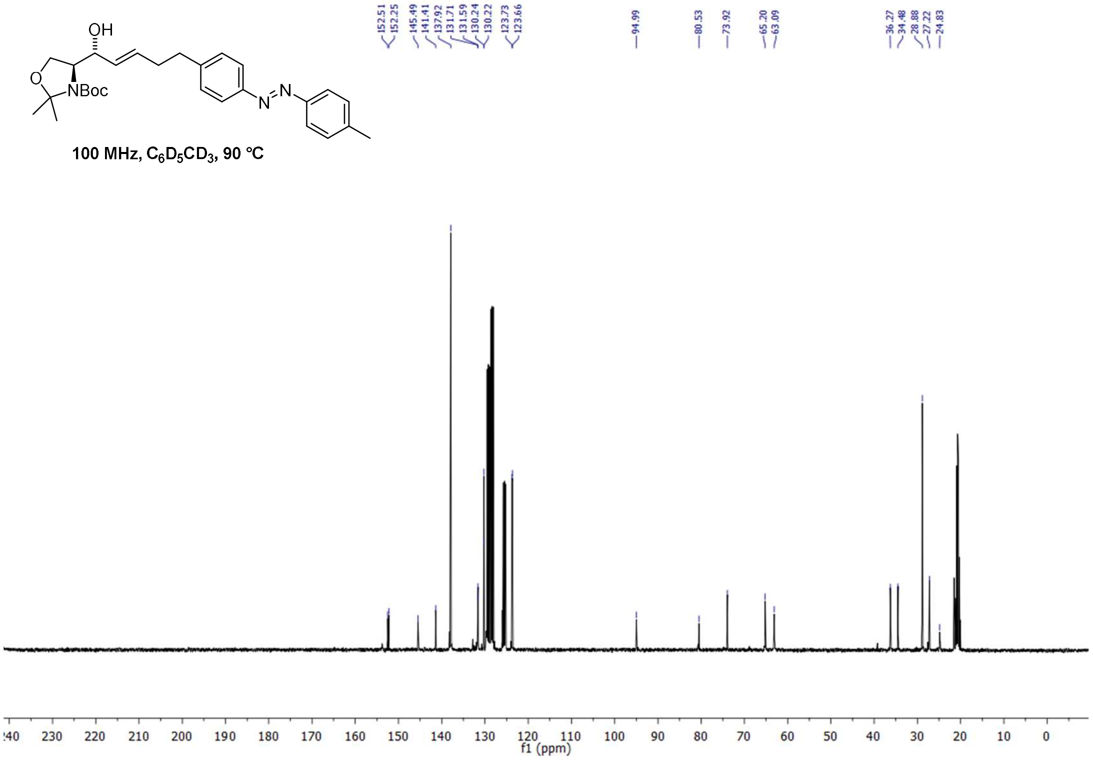

^{1}H NMR of **(2*S*,3*R*,*E*)-2-amino-7-(4-(*p*-tolyldiazenyl)phenyl)hept-4-ene-1,3-diol (aSph-2)**

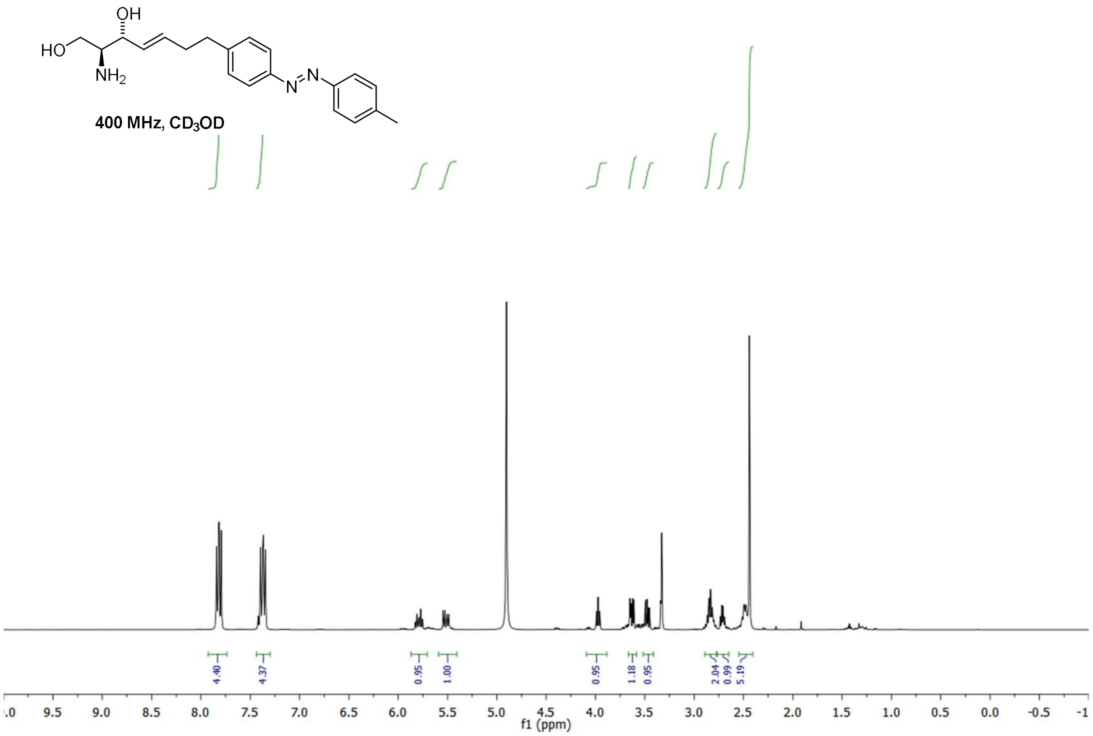

^{13}C NMR of **(2*S*,3*R*,*E*)-2-amino-7-(4-(*p*-tolyldiazenyl)phenyl)hept-4-ene-1,3-diol (aSph-2)**

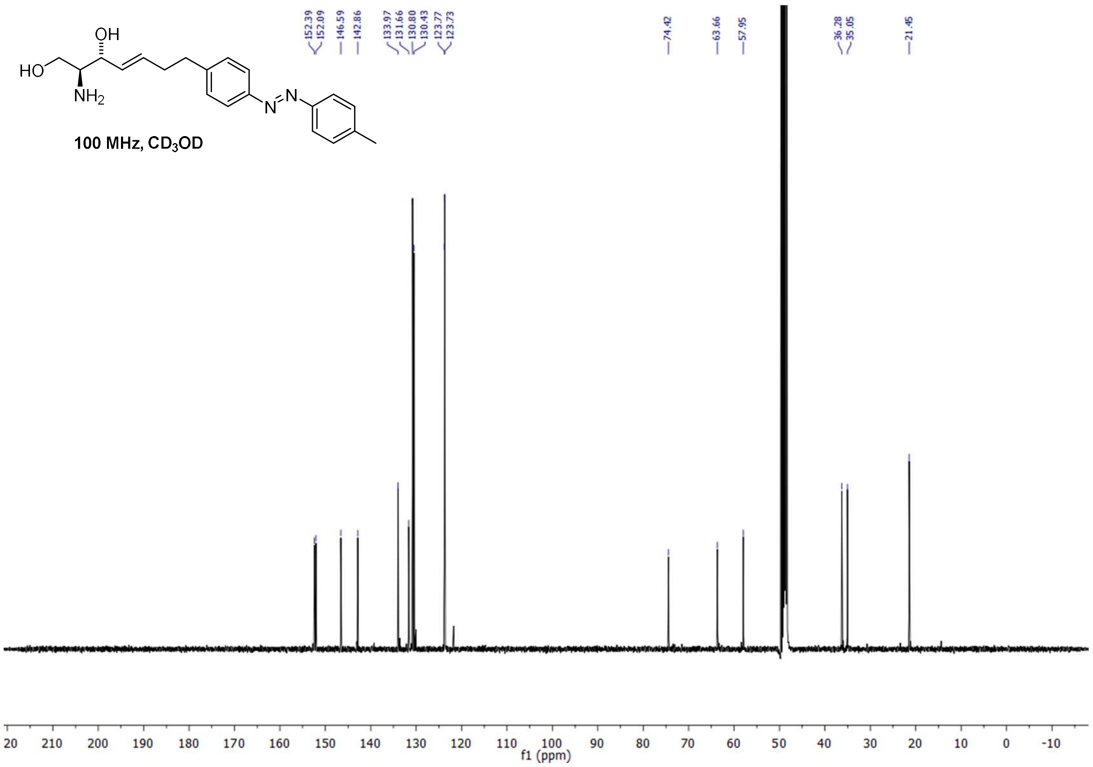

^{1}H NMR of ***N*-((2*S*,3*R*,*E*)-1,3-dihydroxy-7-(4-(*p*-tolyldiazenyl) phenyl)hept-4-en-2-yl)pentadec-14-ynamide (caCer-4)**

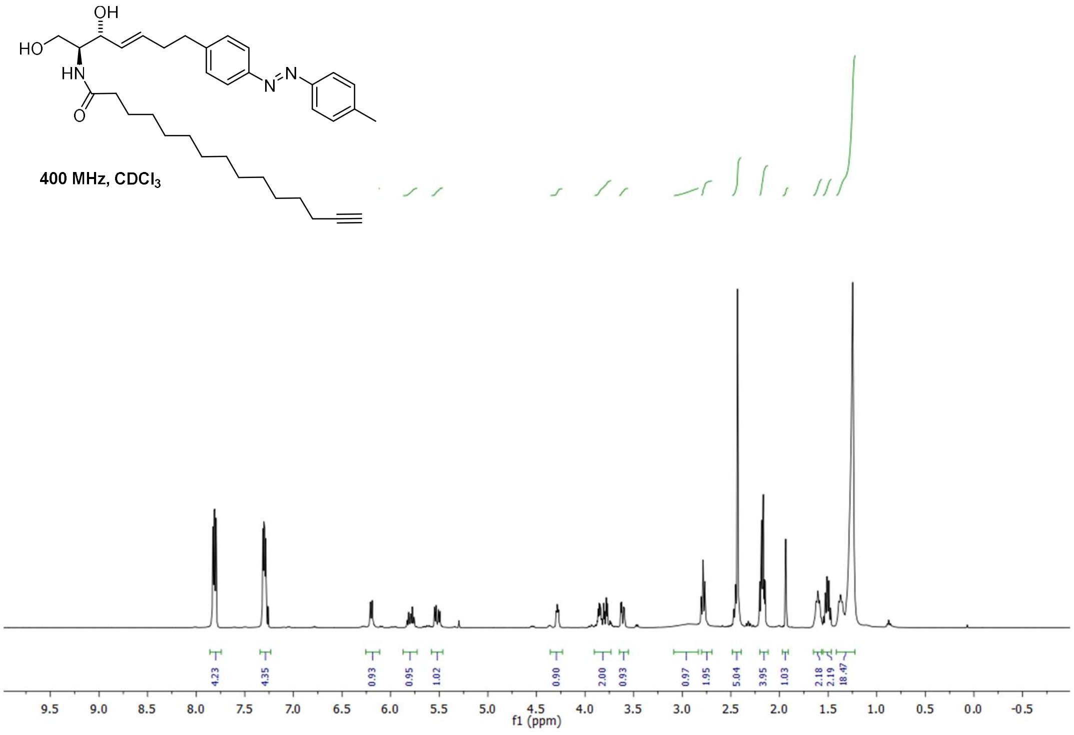

^{13}C NMR of ***N*-((2*S*,3*R*,*E*)-1,3-dihydroxy-7-(4-(*p*-tolyldiazenyl) phenyl)hept-4-en-2-yl)pentadec-14-ynamide (caCer-4)**

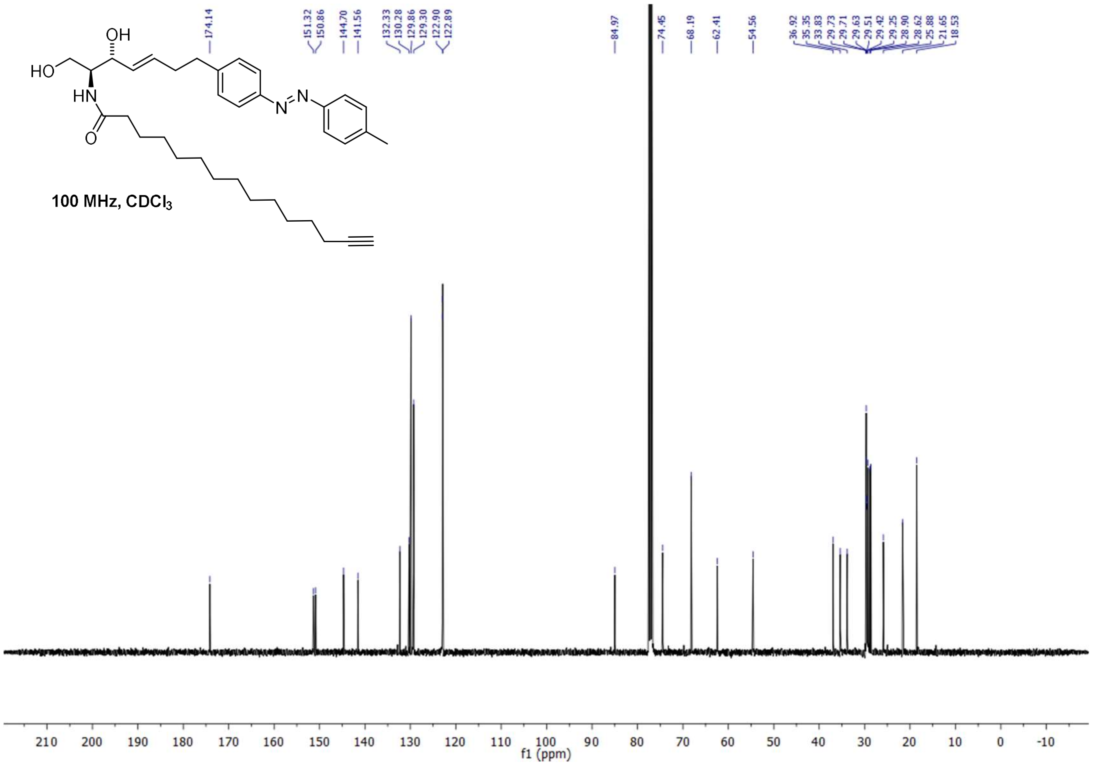

Supplement: Supplementary file 1. [file elife-43230-supp1.pdf]
